# Novel transcriptional regulation of the GAP promoter in *Pichia pastoris* towards high expression of heterologous proteins

**DOI:** 10.1186/s12934-024-02435-9

**Published:** 2024-07-24

**Authors:** Xiangna Lin, Weiqiu Ding, Shaoyan Zheng, Lianna Wu, Xue Chen, Chunfang Xie, Daling Liu, Dongsheng Yao

**Affiliations:** 1 Institute of Biomedicine, Jinan University, Guangzhou City, 510632 Guangdong Province China; 2 National Engineering Research Center of Genetic Medicine, Jinan University, Guangzhou City, 510632 Guangdong Province China; 3 Guangdong Provincial Key Laboratory of Bioengineering Medicine, Jinan University, Guangzhou City, 510632 Guangdong Province China; 4 Department of Bioengineering, Jinan University, Guangzhou City, 510632 Guangdong Province China

**Keywords:** Constitutive expression, pGAP, *P. pastoris*, Transcription factor, Heterologous protein

## Abstract

**Background:**

*Pichia pastoris* (*Komagataella phaffii*) is a promising production host, but the usage of methanol limits its application in the medicine and food industries.

**Results:**

To improve the constitutive expression of heterologous proteins in *P. pastoris,* four new potential transcription regulators (Loc1p, Msn2p, Gsm1p, Hot1p) of the glyceraldehyde triphosphate dehydrogenase promoter (pGAP) were revealed in this study by using *cellulase E4* as reporter gene. On this basis, a series of *P. pastoris* strains with knockout or overexpression of transcription factors were constructed and the deletion of transcription factor binding sites on pGAP was confirmed. The results showed that Loc1p and Msn2p can inhibit the activity of pGAP, while Gsm1p and Hot1p can enhance the activity of pGAP; Loc1p, Gsm1p and Hot1p can bind directly to pGAP, while Msn2p must be treated to expose the C-terminal domain to bind to pGAP. Moreover, manipulating a single transcription factor led to a 0.96-fold to 2.43-fold increase in xylanase expression. In another model protein, aflatoxin oxidase, knocking out *Loc1* based on *AFO-∆Msn2* strain resulted in a 0.63-fold to 1.4-fold increase in expression. It can be demonstrated that the combined use of transcription factors can further improve the expression of exogenous proteins in *P. pastoris*.

**Conclusion:**

These findings will contribute to the construction of pGAP-based *P. pastoris* systems towards high expression of heterologous proteins, hence improving the application potential of yeast.

**Supplementary Information:**

The online version contains supplementary material available at 10.1186/s12934-024-02435-9.

## Background

The *Pichia pastoris* expression system is among the most widely used eukaryotic recombinant protein expression systems. More than 5000 recombinant proteins have been successfully expressed in *P. pastoris* (http://www.pichia.com) [1]. Because it has the general properties of yeast and offers some advantages that other expression systems do not, the *P. pastoris* expression system could become an attractive platform for the expression of foreign proteins [2–6]. One of the most prominent features of this system is the presence of pAOX1 (alcohol oxidase I promoter), a strong and strictly regulated methanol-inducible promoter that is commonly used to drive exogenous gene expression [2]. However, the pAOX1-based *P. pastoris* expression system faces many challenges in heterologous protein expression and production. For example, the presence of carbon source (such as glucose and glycerol) repression limits methanol-induced expression of pAOX1 [7–11]. Additionally, the toxic, flammable, and explosive properties of methanol pose potential safety hazards in storage, transportation, and fermentation processes [2]. Finally, methanol as a carbon source requires a large amount of oxygen during fermentation, which can cause product degradation and make purification more difficult [12]. These factors limit the application of pAOX1-based *P. pastoris* expression systems in the medicine, food, and feed industries.

In view of the drawbacks of using methanol for the induction of pAOX1, the optimization of the *P. pastoris* expression system has received considerable research attention in recent years. Most researches are dedicated to modifying pAOX1 based on its regulatory mechanism through deleting or inserting of cis-acting elements on pAOX1, as well as point mutation of the 5'UTR or core promoter region, etc. [13–20]. However, these modifications failed in eliminating the inhibition caused by high levels of alternative carbon sources such as glucose and glycerol, and were far from reaching the level of industrial application. Another research direction is to develop higher-expression promoters to replace pAOX1 [2, 21–26], including inducible promoters pDAS (Dihydroxyacetone synthase), pFLD1 (Formaldehyde dehydrogenase 1), constitutive promoters pGAP, pTEF1 (Translation extension factor 1) and pGCW14 (Potential glycosyl phosphatidyl inositol (GPI)-anchored protein). Nonetheless, they have not been widely promoted in applications due to unknown regulatory mechanisms, toxic and combustible inducers, or unstable expression. Compared with pAOX1, pGAP is a commonly used constitutive promoter, but its transcriptional regulation has rarely been reported. Qin et al. constructed a GAP promoter library by random mutations (introduced using the error-prone PCR technique), which increased GAP promoter activity [27]. Ata et al. reported that rhGH-producing strains were developed using promoter variants constructed by the targeted deletion or replication of transcription factor-binding sites (TFBSs) [28]. These studies, however, did not reveal the underlying regulatory mechanism.

In this study, transcriptome analysis was performed on a highly expressed strain obtained by directed evolution to screen transcription factors with significant changes. Database prediction and molecular docking were used to further investigate the transcription factor binding sites of pGAP. The newly discovered transcription factors involved in the regulation of the pGAP promoter were confirmed through deletion and overexpression, as well as the modification of the pGAP promoter. During the experiment, it was discovered that the regulation of the four transcription factors is broadly applicable to the expression of heterologous proteins in *P. pastoris*. The combination of transcription factors can result in a higher level of heterologous protein expression. The results of this study provide an important theoretical basis for the construction of *P. pastoris* showing high levels of heterologous protein expression.

## Materials and methods

### Strains, plasmids, enzymes, reagents, and primers

*P. pastoris SMD1168* and *Escherichia coli DH5α/BL21(DE3)*, ppic3.5 k, and pET28a plasmids were purchased from Invitrogen (USA). The pBAN plasmid (GenBank accession no. KF806603), *cellulase E4*(GenBank accession no. L20093.1), *xylanase xynB* (GenBank accession no. JX560731.1) and *aflatoxin oxidase AFO* (GenBank accession No. AY941095.1) were available in our laboratory. Q5™ High-Fidelity DNA Polymerase, T4 DNA ligase, restriction endonucleases, and DNA purification kits were purchased from New England Biolabs (NEB, UK). Plasmid isolation kits were purchased from Tiangen Biotech Co. Ltd. (Beijing, China). Yeast genome extraction kits were purchased from Biotechnology (China). Electrophoretic reagents were purchased from Bio-Rad (USA). The electrophoretic mobility shift assay (EMSA) kit was purchased from Biyuntian (Beijing, China). All other chemicals were of analytical grade. The primers (Table S1) and genes used in this study were synthesized by Shanghai Jierui Bioengineering Company (China).

### Construction and directed evolution of strains expressing E4 cellulase

The "BBPB Biobrick" used as the target gene expression vector was constructed in our laboratory (Figure S1). The expression vector was linearized by digestion using restriction enzymes and transformed into *P. pastoris SMD1168* competent cells by electroporation. Recombinant positive clones were screened on minimal dextrose medium (MD medium) at 28 °C for 72 h.

The screened E4 cellulase-expressing strain (*EX*_*6*_) was subjected to multiple rounds of UV mutagenesis and directed evolution. A strain with a high level of heterologous protein expression was finally obtained (labeled as *EX*_*6-34–16-15*_). See Supporting information 13 for specific steps. Glycerol was used as a carbon source for heterologous protein constitutive production by pGAP promoter, while methanol was used for inducible expression by pAOX1 promoter. The *E4 cellulase* and pGAP promoter of strain *EX*_*6-34–16-15*_ were sequenced, and only the E4 gene was mutated. The *E4 cellulase* subjected to UV mutagenesis was cloned to reconstruct the E4 cellulase expression strain as shown in Figure S1 to verify whether the increase of E4 cellulase expression level was caused by the mutation of *E4* gene (denoted as *uvEX*, including *uvEX*_*2*_*, uvEX*_*4*_*, uvEX*_*5*_).

The culture conditions for the production of several reporter proteins in yeast and the determination of relative amounts were displayed in Supporting information 14.

### Transcription factors screening

*EX*_*6*_ and *EX*_*6-34–16-15*_ strains (marked as *EX*_*6-15*_ in sequencing) were inoculated (1% inoculum concentration) in 200 mL YPG (Yeast Peptone Glycerol) medium and incubated at 28 °C and 200 rpm until the cell culture attained the logarithmic growth phase. Three samples were prepared in parallel. RNA extraction and primary analysis of transcriptome sequence (sequencing conducted using RNA-Seq method) were performed by Suzhou Jinweizhi Biotechnology Co. Ltd. (China).

The TFBSs of the pGAP sequence from *P. pastoris SMD1168* were predicted using the YEASTRACT (http://www.yeastract.com/) web server. For comparison of the identified transcription factors, *S. cerevisiae* was employed as the source species (Figure S2). The UniProt database was used to identify transcriptional regulatory genes in *P. pastoris* that were homologous to *S. cerevisiae* genes, and potential transcriptional regulatory genes with significant differences in expression levels were chosen for experimental verification (differential gene expression level more than two times and q value ≤ 0.05). Simultaneously, HDOCK (http://hdock.phys.hust.edu.cn/) was used to determine if the selected transcription factors could interact with pGAP (Table S2).

The protein model used in molecular docking was obtained by alphafold modeling as shown in Figure S3 (Hot1p: C4QW90; Msn2p: C4R1J8; Gsm1p: C4R1K8; Loc1p: C4QX18). pGAP's 3D model was simulated using the Discovery studio 4.5 software.

### Effects of knockout or overexpression of transcription factors on the function of pGAP

Construction of transcription factor-overexpressing strains: Primers were constructed to amplify transcription factor genes from the *P. pastoris* genome. The overexpression vectors were constructed using pGAP as the promoter (Figure S4). Electroporation was used to transform the linearized plasmid DNA into strain *EX*_*6*_.

Construction of transcription factor knockout strains: Using the mutant strain's genome as a template, primers were built to amplify the upstream and downstream sequences of the transcription factor genes. The *kanamycin* (*Kan*) gene was employed as a tag to build a knockout vector (Figure S5). Electroporation was employed to convert the homologous recombinant segments used for knockout into strain *EX*_*6*_.

OD_600_ was examined at regular intervals to obtain the growth curve of each strain in order to determine whether the knockout or overexpression of transcription factors affected the strains' growth. Each experiment was carried out three times.

Real-time fluorescence quantitative PCR (qPCR) was used to examine the transcription level of *E4*. RNA was isolated from the logarithmic-growing fungus. As a template, cDNA produced through reverse transcription was used. TaKaRa's SYBR Primix Ex TaqII and primers 1-F and 1-R were used for qPCR amplification; the *gapdh* was used as the internal reference gene. Each experiment was carried out three times.

The same biomass strain's culture supernatant was exposed to western blot verification, and protein expression levels were compared.

### Interaction between GAP promoter and transcription factors

The plasmid pET-28a ( +) was used as the expression vector, and *BL21 (DE3)* was used as the host strain. *E. coli* harboring transcription factor expression vectors was used to obtain transcription factors (Figure S6). EMSA was used to investigate the relationships between transcription factors and pGAP. The experimental groups are shown in the Table S3. Each group was incubated for 20 min before the proteins electrophoretically transferred to the membranes at low temperatures. A chemiluminescence imaging system was used for exposure imaging.

### Determination of transcription factor-binding regions on pGAP

To precisely pinpoint the transcription factor binding site on pGAP, it was split into three fragments (Figure S7), dubbed pGAP-AB, pGAP-BC, and pGAP-CD (about 50 bp overlap between two fragments). Furthermore, pGAP (del) was assigned to the nucleic acid probe with two deleted overlapping areas. EMSA was used to examine transcription factor binding sites on pGAP.

### Identification of DNA-binding domains of transcription factors

The SMART software predicted each transcription factor’s DNA-binding domains, transcriptional activation domains, and special junction domains (Table S4). Loc1p, Gsm1p, and Hot1p were partitioned and expressed in *E. coli* based on the position of the domain in the transcription factor sequence; the partial domains were called Loc1p-N and Loc1p-C, Gsm1p-N and Gsm1p-C, and Hot1p1-N and Hot1p-C, respectively. Msn2p’s C-terminus was predicted to have a zinc finger structure, so 100 amino acids at the C-terminus were produced and called Msn2p-C100 (Figure S6). EMSA tests were carried out using the various domains of the four transcription factors as well as the pGAP.

The nuclear localization signal (NLS) of Msn2p was predicted using the online servers, NLStradamus (http://www.moseslab.csb.utoronto.ca/NLStradamus/) [29], NLS Mapper (https://nls-mapper.iab.keio.ac.jp/cgi-bin/NLS_Mapper_form.cgi) [30], and PSORTII (https://www.genscript.com/psort.html) [31] as shown in Figure S8.

### Analysis of pGAP after deletion of TFBSs

According to the software's predictions, putative TFBSs on pGAP were removed to create a series of strains with mutant pGAP sequences (Table 1). Confocal microscopy was used to measure the intensity of the reporter gene's expression (red fluorescent protein, RFP). The activity of the mutant and intact pGAP was compared. Msn2p-C100 was expressed in the *pGAP-rfp-ppic3.5 k-∆Msn2-SMD1168* strain to confirm its inhibitory action. A laser confocal microscope was used for the observation.

**Table 1:** List of strains

Promoter of glyceraldehyde triphosphate dehydrogenase (pGAP) mutant strain	Transcription factor knockout strains	Transcription factor knockout strains with mutant pGAP	Predicted binding sites
*pGAP(∆Loc1)-rfp-ppic3.5 k-SMD1168*	*pGAP -rfp-ppic3.5 k-∆Loc1-SMD1168*	*pGAP(∆Loc1)-rfp-ppic3.5 k∆Loc1-SMD1168*	38–42 bp
*pGAP(∆Msn2)-rfp-ppic3.5 k-SMD1168*	*pGAP -rfp-ppic3.5 k-∆Msn2-SMD1168*		229–234 bp
*pGAP(∆Gsm1)-rfp-ppic3.5 k-SMD1168*	*pGAP -rfp-ppic3.5 k-∆Gsm1-SMD1168*	*pGAP(∆Hot1)rfp-ppic3.5 k-∆Hot1-SMD1168*	70–82 bp
*pGAP(∆Hot1)-rfp-ppic3.5 k-SMD1168*	*pGAP -rfp-ppic3.5 k-∆Hot1-SMD1168*		158–162 bp, 168–172 bp, 247–251 bp, 305–309 bp, 452–459 bp

### Validation of applicability of transcription factors

To further validate the regulatory effect of these four transcription factors on the overexpression of heterologous proteins in *P. pastoris* using pGAP as the promoter, other heterologous protein expression strains were constructed. Firstly, the activating and inhibitory transcription factors were overexpressed and knocked out in the *P. pastoris* strain expressing xyn B(xylanase), respectively. Furthermore, a single transcription inhibitor was knocked out in the strain expressing AFO (aflatoxin oxidase). On this basis, another transcription inhibitor was knocked out to form a double transcription factor knockout strain. The effects of single transcription inhibitor knockout and double knockout strains on protein expression were compared.

## Results and discussion

### Directed evolution of strains showing constitutive high level of expression

**Fig. 1:**
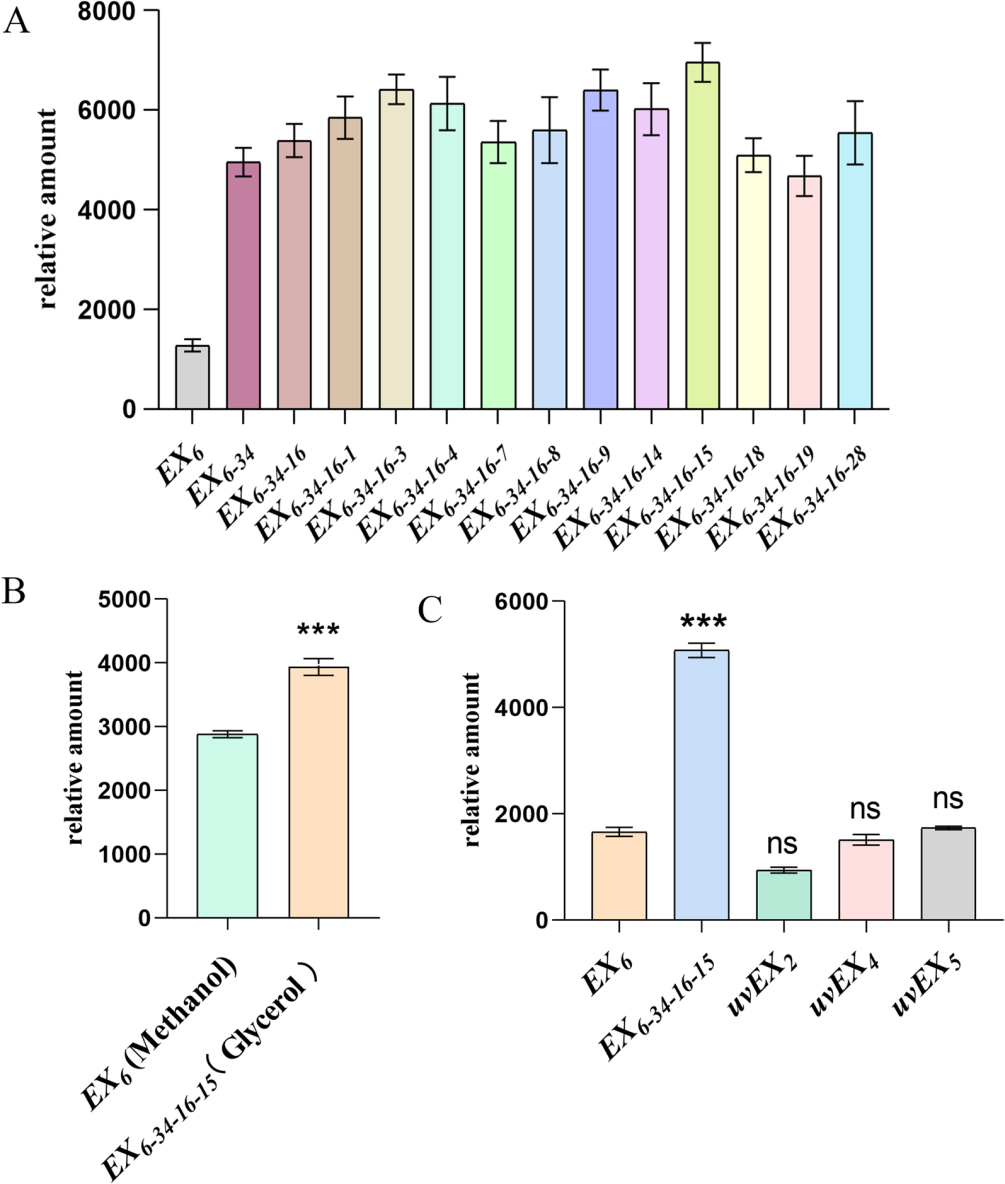
Expression levels of E4 proteins in different *P. pastoris* strains. **A** Levels of protein expression in different strains during directed evolution. **B** Comparison of expression levels of *E*_*4*_ cellulase in *EX*_*6-34–16-15*_ strain (using pGAP promoter and glycerol as carbon source) and *EX*_*6*_ strain (using pAOX1 promoter and methanol as carbon source). **C** Comparison of E4 expression levels in *uvEX, EX*_*6*_*, and EX*_*6-34–16-15*_ strains. *EX*_*6*_ was used as a control. The mean values of the results of three repeated experiments are depicted, and the relative standard deviations are shown using error bars (*p < 0.05, **p < 0.01, ***p < 0.001)

With *E4 cellulase* as reporter gene, *P. pastoris* strain *EX*_*6*_ was treated to directed evolution, obtaining the genetically stable strain *EX*_*6-34–16-15*_, in which the expression of E4 cellulase increased by 444% over *EX*_*6*_. The results are shown in Fig. 1A. Figure 1B shows that the expression level of E4 cellulase in strain *EX*_*6-34–16-15*_ is 1.37 times higher than that in strain *EX*_*6*_*.*

Sequencing of the E4 cellulase gene and pGAP promoter of train *EX*_*6-34–16-15*_ showed no mutations in the pGAP promoter sequence, while mutations occurred in the E4 cellulase gene. Subsequently, the reporter gene (E4 cellulase) from *EX*_*6-34–16-15*_ was used to construct a recombinant expression of E4 cellulase in *P. pastoris* (*uvEX*_*2*_, *uvEX*_*4*_*, uvEX*_*5*_). The expression level of E4 cellulase was not significantly different from that of *EX*_*6*_ (Fig. 1C).

These results suggest that the high expression of E4 cellulase in *EX*_*6-34–16-15*_ may be due to changes in pGAP regulation. This provides a basis for further studying the regulatory mechanism of pGAP through transcriptional differential analysis.

### Analysis of TFBSs in the pGAP sequence

Putative TFBSs in the pGAP sequence were predicted using the YEASTRACT database. Transcription factors homologous to *Saccharomyces cerevisiae* were identified based on the degree of significance of differences in gene expression levels observed in the transcriptome sequence data.

The String database (https://cn.string-db.org/) discovered that Loc1p, a transcription factor with significantly downregulated expression interacted with Ash1p during the screening of potential transcriptional regulatory genes (Figure S9). Because Ash1p was anticipated to have binding sites on pGAP, the interaction sites of Loc1p and pGAP were discovered to overlap with the interaction sites of Ash1p predicted by YEASTRACT via molecular docking. As a result, Loc1p is among the prospective regulating genes for biological research. The results showed that four unreported transcription factors, Loc1p, Msn2p, Gsm1p and Hot1p, may regulate the activity of pGAP (Table 2).

**Table 2:** Prediction of transcription factors

*P. pastoris* transcription factor	Predicted role	Predicted transcription factor binding sites and frequency of occurrence	Homologous protein (*Saccharomyces cerevisiae*)	Regulatory effect (*Saccharomyces cerevisiae*)
Loc1p (PAS_chr1-1_0414)	Inhibition	YTGAT (2)	Loc1p	1. Effective localization of ASH1 mRNA is required [32] 2. Regulation of transcription of other genes [33]
Msn2p (PAS_chr2-1_0723)	Inhibition	AGGGG,CCCCT, RGGGG (2)	Msn2p	Improves cell sensitivity and response to environmental stresses [34]
Gsm1p (PAS_chr2-1_0732)	Activation	CGGNNNNNNNNCGG (1)	Gsm1p	1. Expression of regulatory protein OXPHOS [35] 2. Transcriptional regulation of genes involved in gluconeogenesis pathway [36]
Hot1p (PAS_chr1-1_0149)	Activation	GGGACAAA, CTTCC,CWTCC (5)	Gcr1p	1. Involved in glycolysis to promote cell growth [37] 2. Involved in maintaining the stability of cellular inositol levels, maintaining the structure of vacuoles, and promoting cell autophagy and renewal [38]

Loc1p owned two relatively concentrated binding sites, one of which coincides with the binding site of its interacting protein Ash1p, according to HDOCK. Therefore, Loc1p was regarded to have a binding site comparable to Ash1p (Table S2). Thus, investigating the relationship between these transcription factors and pGAP in *P. pastoris* would aid in understanding the mechanism of pGAP activity regulation.

### Knockout and overexpression of transcription factors

**Fig. 2:**
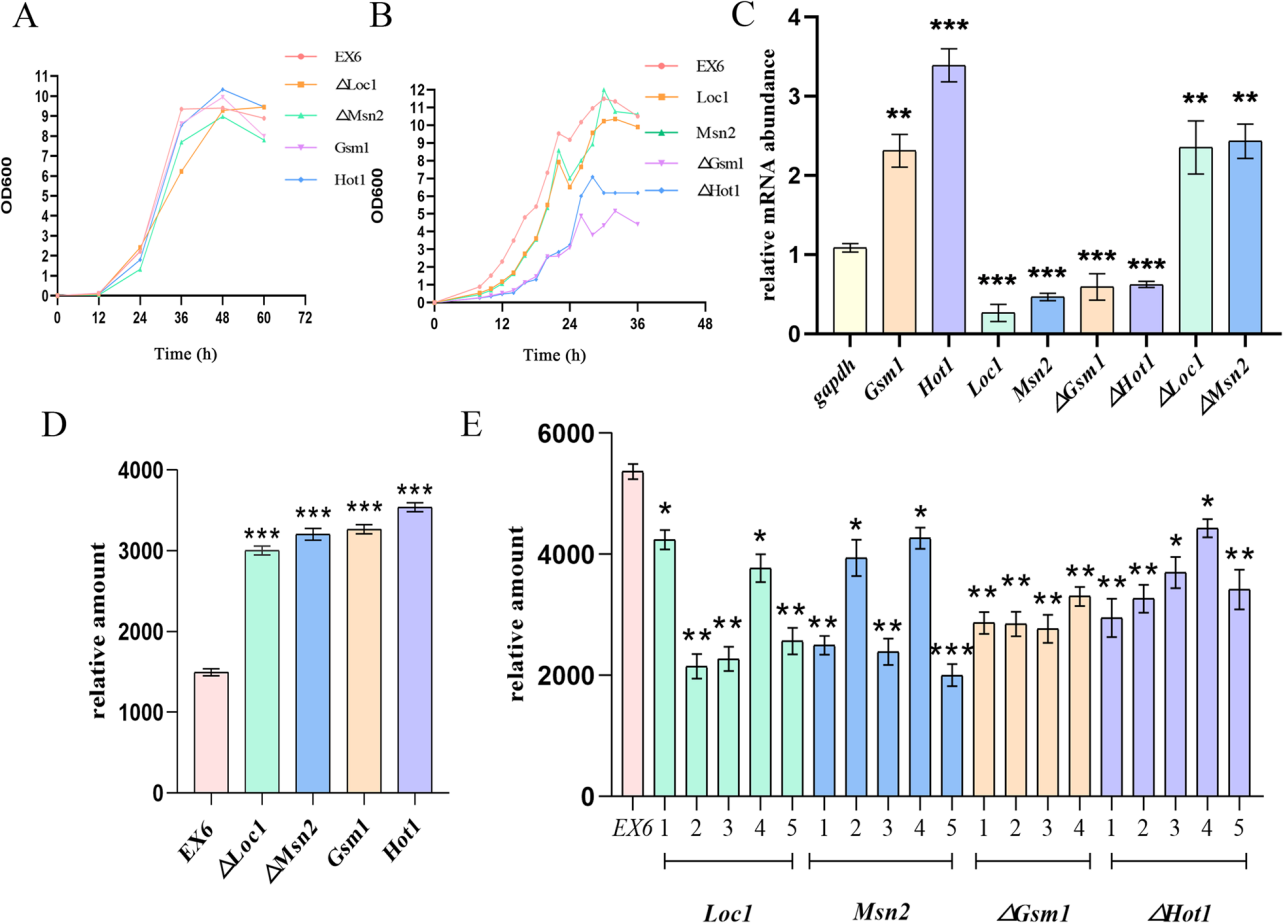
Growth, gene transcription, and protein expression in different strains of *P. pastoris*. (**A**–**B**) Growth curves of different strains. Strains in (**A**) had increased expression levels; strains in (**B**) had decreased expression levels. **C** Quantitative real-time PCR (qPCR) results: Taking *gapdh* as the control, the average values of the results of three repeated experiments were depicted and the relative standard deviations are shown using error bars. **D**–**E** Western blotting (WB) results: Taking *EX*_*6*_ as the control, Strains in (**D**) had increased expression levels; strains in (**E**) had decreased expression levels. The numbers 1 to 5 indicate 5 different clones of the strain. The average values of the results of three repeated experiments were depicted, and the relative standard deviations are shown using error bars. Protein expression levels in each strain were measured under the same biomass conditions (*p < 0.05, **p < 0.01, ***p < 0.001)

The growth curve showed that the knockout of *Hot1* and *Gsm1* reduced the growth rates of the strains by about 20%-30%, while the overexpression of *Hot1* had a growth rate 1.1 times that of the *EX*_*6*_. The overexpression and knockout of *Loc1* and *Msn2* had no effect on the growth rates of the strains (Fig. 2A to B). Hot1p and Gsm1p are involved in the regulation of glycolysis and gluconeogenesis in *S. cerevisiae*, according to Ravi et al. [38]. The knockout of *Hot1* and *Gsm1* may inhibit cell growth by disrupting cellular metabolism.

The levels of *E4* gene transcription and E4 expression in *EX*_*6*_ strains, in which the putative transcription factors were overexpressed and knocked out, are shown in Fig. 2 C to 2E. Compared to the strain *EX*_*6*_, the levels of *E4* gene transcription and E4 expression in *∆Loc1, ∆Msn2, Gsm1*, and *Hot1* strains were significantly upregulated, but they were significantly downregulated in *Loc1, Msn2, ∆Gsm1*, and *∆Hot1* strains. The results of WB and qPCR analyses corroborated these findings. Specifically, Hot1p and Gsm1p were identified to enhance the function of the GAP promoter, whereas Loc1p and Msn2p had the opposite effect. This suggested that these four transcription factors play a role in regulating pGAP activity, as their inhibition or activation resulted in a corresponding change in pGAP activity levels.

### Interaction between GAP promoter and transcription factors

**Fig. 3:**
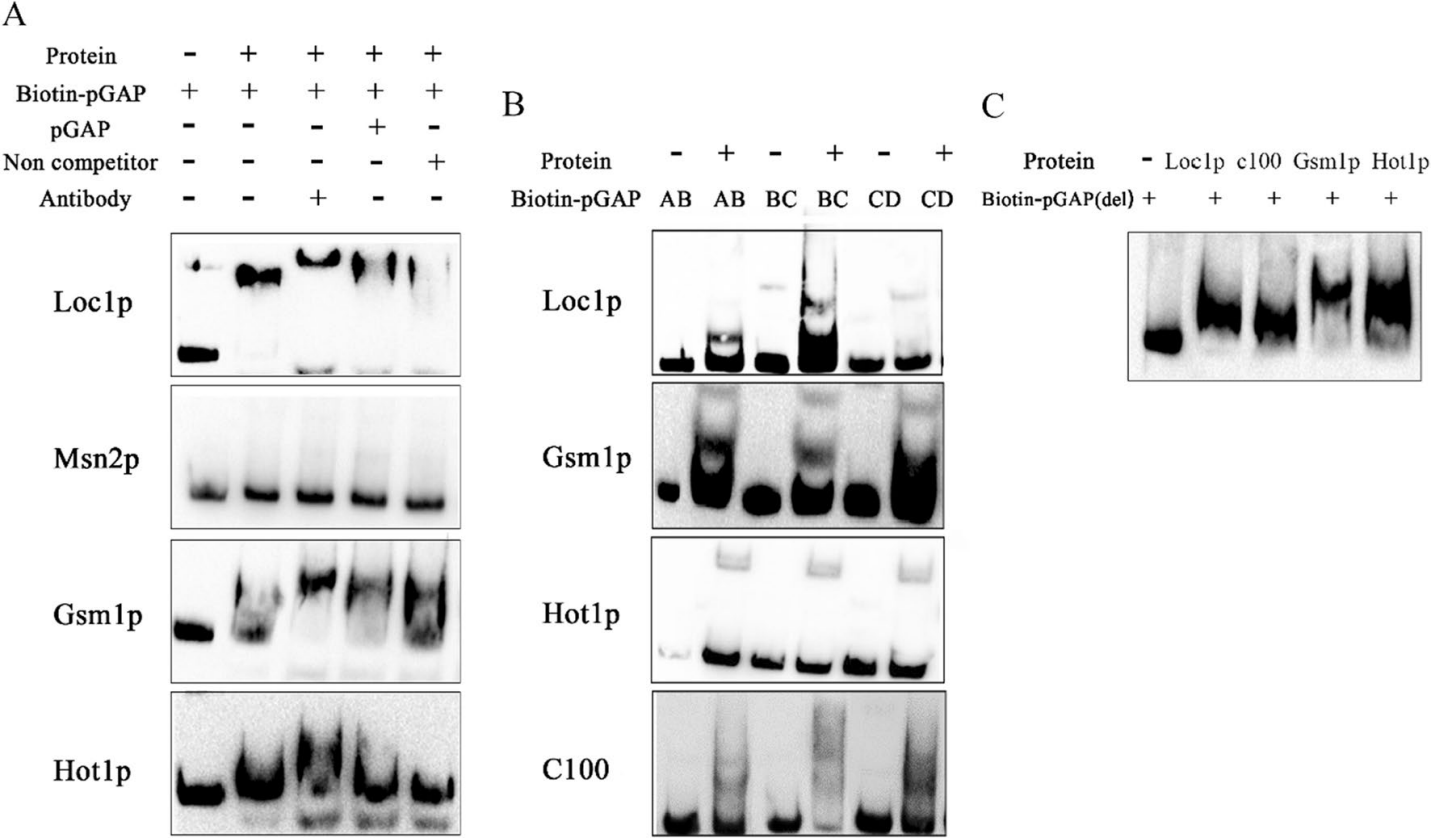
EMSA results showing interactions between transcription factors and promoters of glyceraldehyde triphosphate dehydrogenase (pGAP) fragments. **A** EMSA results showing interactions between the four transcription factors and the full-length pGAP. **B** EMSA results showing interactions between the transcription factors and the partial fragments of pGAP. **C** EMSA results showing interactions between the transcription factors and the pGAP (del) fragments. The “ + ” (“ − ”) sign indicates that the corresponding components were added (not added)

The EMSA results showed that Loc1p, Gsm1p, and Hot1p could specifically bind to pGAP (Fig. 3A), indicating that these transcription factors could directly bind to pGAP and regulate its activity. The binding of Msn2p with pGAP was not detected in the in vitro experiments. However, the zinc finger domain of Msn2p, composed of 100 amino acids at its C-terminus (Msn2p-C100), could bind to pGAP (Fig. 3A).

In this study, EMSA was performed to analyze the interaction of the four regulatory factors with three pGAP segments, and the results are shown in Fig. 3B. Hot1p, Gsm1p, and Msn2p-C100 interacted with all three segments of pGAP, while Loc1p interacted with the pGAP-AB and pGAP-BC. To determine whether the binding site was present in the overlapping regions of the segments, EMSA was conducted to analyze the binding of the transcriptional regulators with pGAP (del), from which the overlapping region of the promoter sequence was deleted. Loc1p, C100, Gsm1p and Hot1p were found to bind to pGAP (del) (Fig. 3C). These results suggested that there might be multiple TFBSs in pGAP.

However, the positions of these TFBSs were different those of TFBSs in pGAP predicted by YEASTRACT. HDOCK was used to examine the docking of transcription factors (Table S2). The sites where Loc1p interacted with pGAP were mainly concentrated in the pGAP-AB and pGAP-BC segments, and these sites were ranked among the top 10 positions where binding was predicted. The YTGAT site was included in the main binding site of pGAP-AB, indicating that the binding site of Loc1p did overlap with ' YTGAT '. However, ' YTGAT ' was only a portion of the Loc1p binding site, and therefore, there was no interaction on pGAP-CD. The sites where Hot1p, Gsm1p, and C100 interacted with pGAP were distributed in all three segments of pGAP. These docking results were consistent with the EMSA. The transcription factors were observed to have multiple putative binding sites on pGAP, and additional investigations are necessary to ascertain the precise localization of these binding sites. This study demonstrates that molecular simulation docking enables an initial exploration of the interaction between transcription factors and promoters, potentially enhancing the efficiency of mining promoter regulatory mechanisms.

**Fig. 4:**
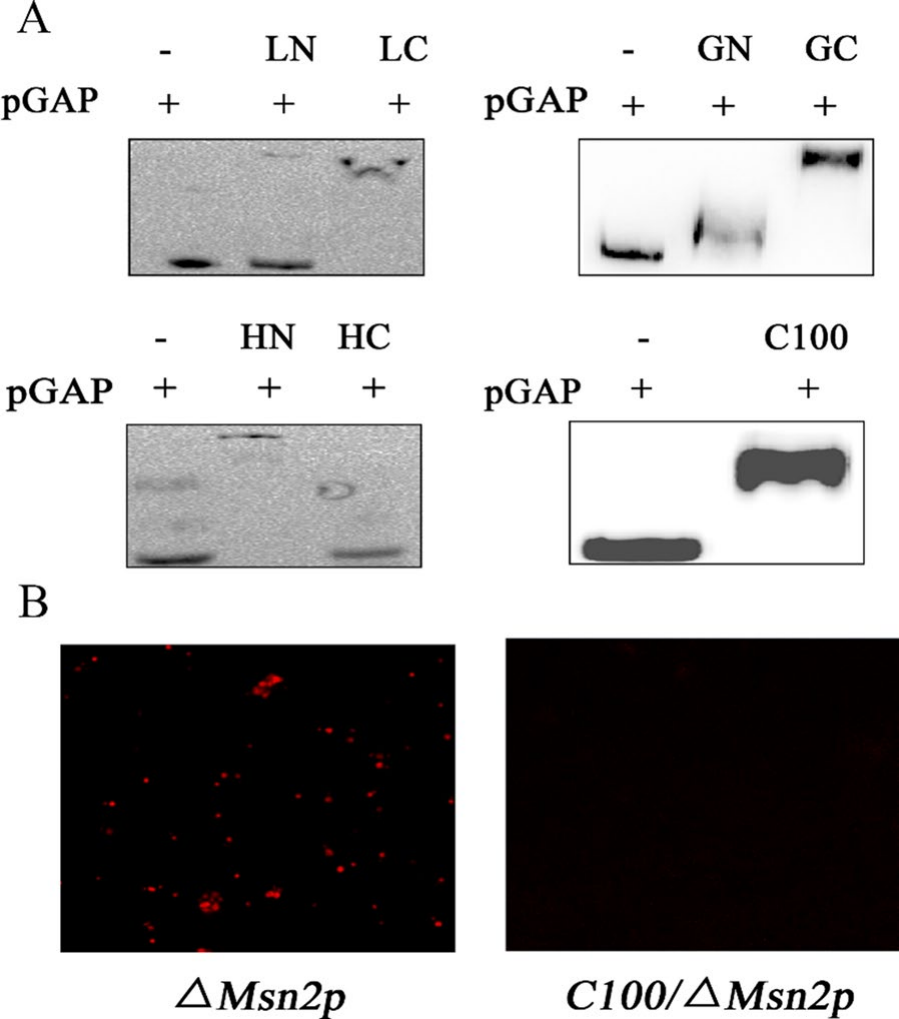
Identification of DNA-binding domains of transcription factors. **A** Determination of DNA-binding domains of the transcription factors. The “ + ” sign indicates that the corresponding components were added; the “ − ” sign indicates that the corresponding components were not added. LN:Loc1p-N; LC:Loc1p-C; GN:Gsm1p-N; GC:Gsm1p-C; HN:Hot1p-N; HC:Hot1p-C; C100:Msn2p-C100. **B** The transcription factor *Msn2*-knockout strain and the C100-complemented strain were analyzed using a laser scanning confocal microscope (20 ×)

The EMSA experiments showed that the Loc1p-C, Gsm1p-N, Gsm1p-C, Hot1p-N, and Msn2p-C100 domains could interact with pGAP, as demonstrated by the presence of blocked bands (Fig. 4A). The Msn2p-C100 replenishment experiment was carried out using the *pGAP-rfp-ppic3.5 k-∆Msn2-SMD1168* strain. A laser confocal microscope was used to examine the reporter gene’s expression. The fluorescence intensity of the Msn2p-C100 replenishment strain was significantly lower than that of the *Msn2* knockout strain (Fig. 4B). According to the experimental results, the binding domain of Loc1p is a randomly coiled structure composed of 113 amino acids at the C-terminus, Gsm1p has multiple binding domains (the zinc finger-like structure at the N-terminus and the presumed active site at the N-terminus), and the binding domain of Hot1p is in a coiled helix composed of 200 amino acids at the N-terminus. Msn2p's binding domain may be a zinc finger domain with 100 amino acids at the C-terminus.

The online servers NLStradamus, NLS Mapper, and PSORT II predicted the nuclear localization signal (NLS) of Msn2p was located at amino acid positions 269 to 280 (Figure S8). Msn2p's regulation mechanism shows that the transcription factor's NLS is concealed under normal cellular circumstances. Under certain conditions, Msn2p is modified or cleaved by various enzymes, and the NLS at the N-terminal of the zinc finger domain (C100) is exposed so that C100 can enter the nucleus under the guidance of NLS and exercise the transcriptional inhibition function. In *S. cerevisiae*, Msn2p responds to various stress conditions and can also be phosphorylated by protein kinase A (PKA) under sufficient carbon conditions, thereby inhibiting its nuclear localization. When the cells are in a state of starvation, Msn2p activates yeast Cip1 along with other transcription factors to inhibit the binding of the cyclin complex at the G1 stage (Cdk1-G1), delaying the cell cycle and preventing cell damage [39]. In this study, the deletion of the *Msn2* enhanced the expression level of the reporter protein, whereas protein expression was inhibited after supplementation with Msn2p-C100. This indicates that Msn2p inhibits the activity of pGAP when sufficient nutrients are available, and this inhibition is mediated by its C-terminal zinc finger domain.

### Analysis of TFBSs in the GAP promoter

**Fig. 5:**
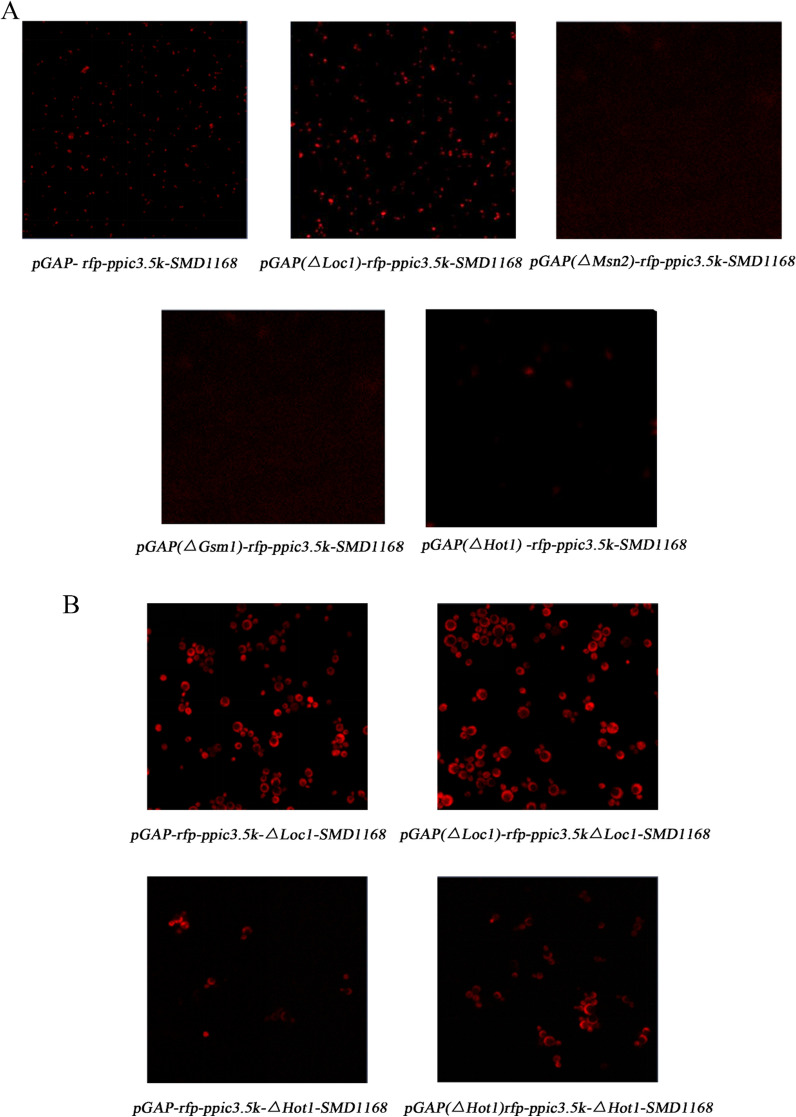
Fluorescence maps of strains harboring mutations in GAP promoter. **A** The results of analysis of pGAP mutant strains using a laser scanning confocal microscope (20 ×). **B** The results of analysis of transcription factor knockout strains harboring mutated pGAP using a laser scanning confocal microscope (40 ×)

The fluorescence intensity of the pGAP strain with a deletion of the Loc1p binding site was considerably higher than that of the strain with the entire pGAP sequence (Fig. 5A). This result showed that the deletion of the binding site alleviated the inhibition of pGAP activity by Loc1p.

However, pGAP strains with deleted Msn2p and Gsm1p binding sites did not emit fluorescence signals, indicating that the deletion of these sites from the pGAP fragment resulted in pGAP inactivation. YEASTRACT analysis of the GAP promoter revealed that there were two Stb5p binding sites in the Gsm1p binding site, while the Msn2p binding site overlapped with the binding sites of multiple transcription factors (Skn7p, Stb5p, Mal63p, Msn4p, Nrg1p, Gis1p, Rph1p, Com2p, Usv1p). An NCBI database search showed that most of these genes promote the activity of DNA-binding transcription factors and RNA polymerase II. Therefore, it is speculated that the loss of Msn2p and Gsm1p binding sites could lead to the loss of important components of pGAP, resulting in the loss of pGAP function.

The fluorescence intensity of *pGAP(∆hot1)-rfp-ppic3.5 k-SMD1168* was significantly weaker than that of *pGAP-rfp-ppic3.5 k-SMD1168*, indicating that deletion of the Hot1p binding site resulted in a decrease in the level of pGAP activation. This result was consistent with the prior finding (section of Knockout and overexpression of transcription factors) that Hot1p is a transcriptional activator of pGAP.

Figure 5B shows that no significant differences were observed in the fluorescence intensities of the *Loc1*-knockout strain (*pGAP-rfp-ppic3.5 k-∆Loc1-SMD1168*) and the Loc1p binding site-knockout strain (*pGAP (∆Loc1)-rfp-ppic3.5 k-∆Loc1-SMD1168*). Likewise, no significant differences were observed in the fluorescence intensities between the Hot1p binding site-knockout strain (*pGAP(∆Hot1)- rfp-ppic3.5 k-∆Hot1-SMD1168*) and the *Hot1*-knockout strain (*pGAP-rfp-ppic3.5 k-∆Hot1-SMD1168*). These results demonstrated that the changes in RFP expression intensities in pGAP mutant strains were caused by the inhibition of transcription factor binding to pGAP rather than shortening of the GAP promoter length. These findings validated the hypothesis that Loc1p is a transcriptional inhibitor of pGAP and Hot1p is a transcriptional activator of pGAP.

### Validation of applicability of transcription factors

**Fig. 6:**
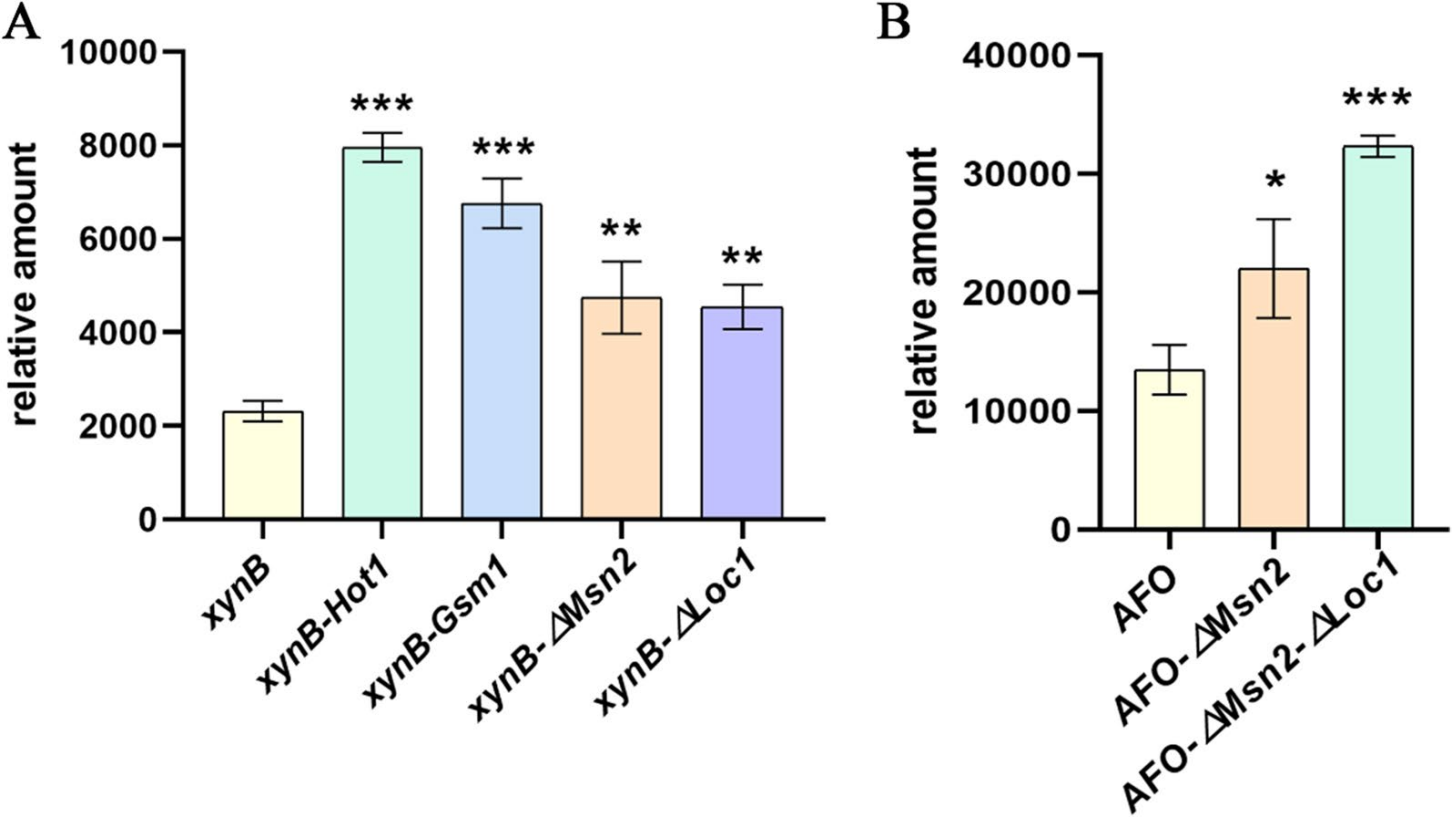
Levels of protein expression in different strains. **A** Comparison of xylanase expression levels in *xynB, xynB-Hot1, xynB-Gsm1, xynB-∆Msn2 and xynB-∆Loc1* strains. *xynB* was used as a control. **B** Comparison of aflatoxin oxidase expression levels in *AFO, AFO-∆Msn2 and AFO-∆Msn2-∆Loc1* strains. *AFO* was used as a control. The mean values of the results of three repeated experiments are depicted, and the relative standard deviations are shown using error bars (*p < 0.05, **p < 0.01, ***p < 0.001)

As shown in Fig. 6A, compared to the *xynB* strain, the expression levels of xynB in the *xynB-Hot1* and *xynB-Gsm1* strains increased by 2.43 and 1.92 times respectively. Moreover, the expression levels of xynB in the *xynB-∆Msn2* strain and the *xynB- ∆Loc1* strain were 2.05 and 1.96 times that of the *xynB* strain. The AFO expression level of the *AFO-∆Msn2* strain increased by 1.63 times. Based on this, further knockout of *Loc1* (*AFO-∆Msn2-∆Loc1* strain) resulted in a 1.4-fold increase in AFO expression (Fig. 6B). The results show that the combination of transcription factors can enhance heterologous protein production further.

**Fig. 7:**
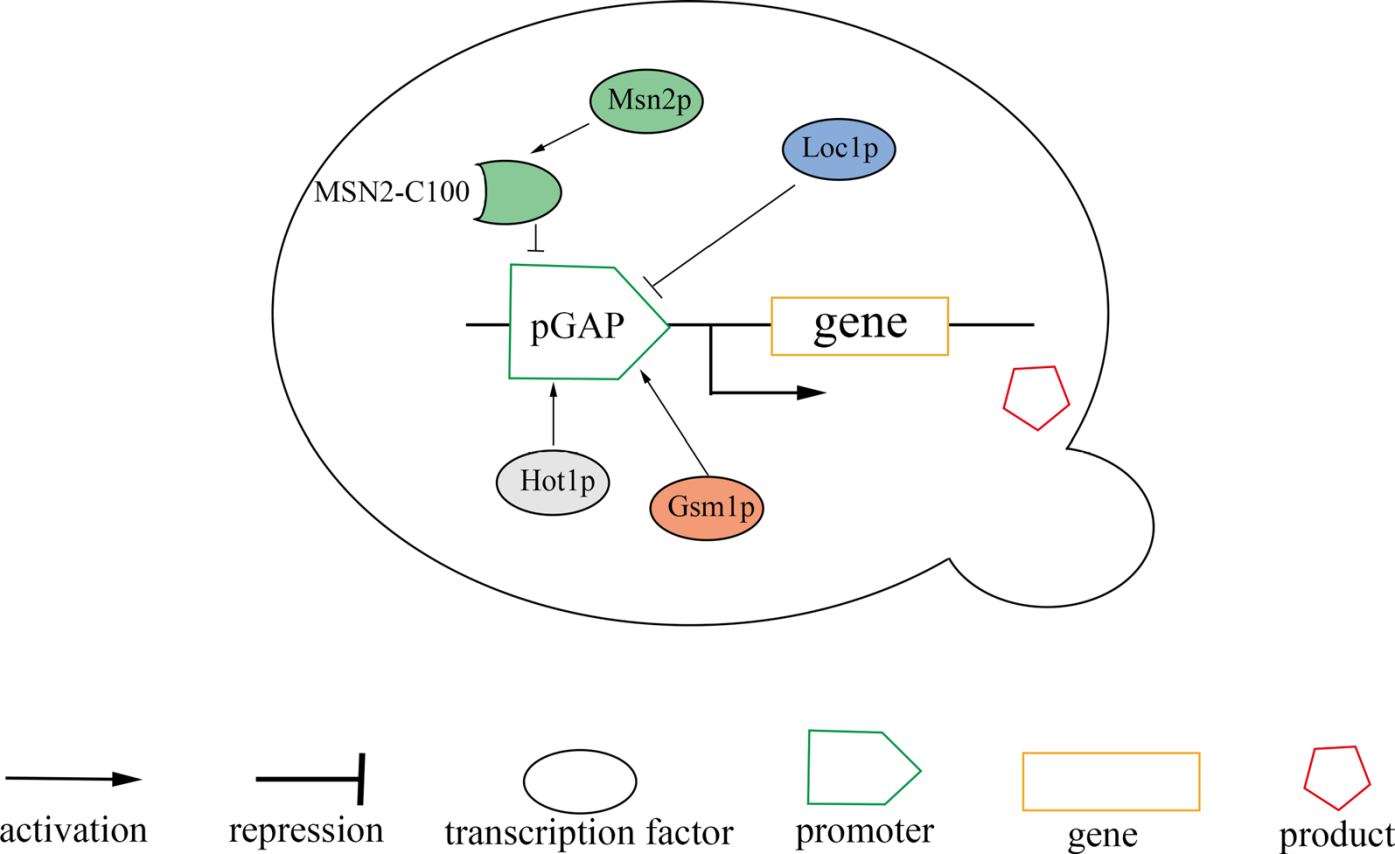
Regulation of pGAP activity by the four transcription factors (Loc1p, Msn2p, Gsm1p, and Hot1p)

Overall, these findings confirm that these transcription factors participate in the regulation of pGAP activity (Fig. 7).

## Conclusion

The study identified four novel transcription factors (Loc1p, Msn2p, Gsm1p, and Hot1p) that regulate the activity of the GAP promoter. Furthermore, it was demonstrated that Loc1p and Msn2p are transcriptional inhibitors of pGAP, and Gsm1p and Hot1p are transcriptional activators of pGAP. Regulating pGAP with the four transcription factors can lead to increased heterologous protein expression in *P. pastoris*.

The results of this study provide a theoretical basis for the construction of strains with constitutively high expression levels of heterologous proteins. This engineering strategy can also be used for other constitutive promoters to improve the constitutive expression level of heterologous proteins in *P. pastoris*, making it more suitable for production applications in the field of medicine and food technology.

### Supplementary Information

Supplementary material 1.

## Data Availability

The datasets and materials used and/or analysed during the current study are available from the corresponding author on reasonable request.

## Abbreviations

pGAP: Glyceraldehyde triphosphate dehydrogenase promoter
5′ UTR: 5′ Untranscribed region
TFBS: Transcription factor-binding site
EMSA: Electrophoretic mobility shift assay
MD medium: Minimal dextrose medium
NLS: Nuclear localization signal
RFP: Red fluorescent protein
WB: Western blot
qPCR: Quantitative polymerase chain reaction

## References

[1] Cereghino JL, Cregg JM. Heterologous protein expression in the methylotrophic yeast *Pichia pastoris*. FEMS Microbiol Rev. 2000;24:45–66. 10640598 10.1111/j.1574-6976.2000.tb00532.x

[2] Vogl T, Glieder A. Regulation of *Pichia pastoris* promoters and its consequences for protein production. New Biotechnol. 2013;30:385–404. 10.1016/j.nbt.2012.11.010 23165100

[3] De Schutter K, Lin YC, Tiels P, Van Hecke A, Glinka S, Weber-Lehmann J, Rouzé P, Van de Peer Y, Callewaert N. Genome sequence of the recombinant protein production host *Pichia pastoris*. Nat Biotechnol. 2009;27:561. 19465926 10.1038/nbt.1544

[4] Liu WC, Zhu P. Scale high-cell-density fermentation of *Pichia pastoris*. In: Picanço-Castro V, Swiech K, editors. Recombinant glycoprotein production. New York: Springer; 2018. p. 109–16.

[5] Hohenblum H, Gasser B, Maurer M, Borth N, Mattanovich D. Effects of gene dosage, promoters, and substrates on unfolded protein stress of recombinant *Pichia pastoris*. Biotechnol Bioeng. 2004;85:367–75. 14755554 10.1002/bit.10904

[6] Clare J, Rayment F, Ballantine S, Sreekrishna K, Romanos M. High-level expression of tetanus toxin fragment C in *Pichia pastoris* strains containing multiple tandem integrations of the gene. Bio/Technology. 1991;9:455–60. 1367310 10.1038/nbt0591-455

[7] Cregg JM, Madden K, Barringer K, Thill G, Stillman C. Functional characterization of the two alcohol oxidase genes from the yeast *Pichia pastoris*. Mol Cell Biol. 1989;9:1316–23. 2657390 10.1128/mcb.9.3.1316-1323.1989 PMC362724

[8] Heyland J, Fu J, Blank LM, Schmid A. Carbon metabolism limits recombinant protein production in *Pichia pastoris*. Biotechnol Bioeng. 2011;108:1942–53. 21351072 10.1002/bit.23114

[9] Sreekrishna K, Brankamp RG, Kropp KE, Blankenship DT, Tsay J-T, Smith PL, Wierschke JD, Subramaniam A, Birkenberger LA. Strategies for optimal synthesis and secretion of heterologous proteins in the methylotrophic yeast *Pichia pastoris*. Gene. 1997;190:55–62. 9185849 10.1016/S0378-1119(96)00672-5

[10] Chauhan A, Arora D, Khanna N. A novel feeding strategy for enhanced protein production by fed-batch fermentation in recombinant *Pichia pastoris*. Process Biochem. 1999;34:139–45. 10.1016/S0032-9592(98)00080-6

[11] Cos O, Ramón R, Montesinos JL, Valero F. Operational strategies, monitoring and control of heterologous protein production in the methylotrophic yeast *Pichia pastoris* under different promoters: a review. Microb Cell Fact. 2006;5:1–20. 16600031 10.1186/1475-2859-5-17 PMC1564409

[12] Sola A, Jouhten P, Maaheimo H, Sanchez-Ferrando F, Szyperski T, Ferrer P. Metabolic flux profiling of *Pichia pastoris* grown on glycerol/methanol mixtures in chemostat cultures at low and high dilution rates. Microbiology. 2007;153:281–90. 17185557 10.1099/mic.0.29263-0

[13] Inan M. Studies on the alcohol oxidase (AOX1) promoter of *Pichia pastoris*. J Biosci Bioeng. 2002. 10.1016/S1389-1723(01)80321-2. 10.1016/S1389-1723(01)80321-2 16233151

[14] Hartner FS, Ruth C, Langenegger D, Johnson SN, Hyka P, Lin-Cereghino GP, Lin-Cereghino J, Kovar K, Cregg JM, Glieder A. Promoter library designed for fine-tuned gene expression in *Pichia pastoris*. Nucl Acid Res. 2008;36:e76–e76. 10.1093/nar/gkn369 PMC2475614 18539608

[15] Ruth C, Zuellig T, Mellitzer A, Weis R, Looser V, Kovar K, Glieder A. Variable production windows for porcine trypsinogen employing synthetic inducible promoter variants in *Pichia pastoris*. Syst Synth Biol. 2010;4:181–91. 21886682 10.1007/s11693-010-9057-0 PMC2955199

[16] Xuan Y, Zhou X, Zhang W, Zhang X, Song Z, Zhang Y. An upstream activation sequence controls the expression of AOX1 gene in *Pichia pastoris*. FEMS Yeast Res. 2009;9:1271–82. 19788557 10.1111/j.1567-1364.2009.00571.x

[17] Staley CA, Huang A, Nattestad M, Oshiro KT, Ray LE, Mulye T, Li ZH, Le T, Stephens JJ, Gomez SR. Analysis of the 5′ untranslated region (5′ UTR) of the alcohol oxidase 1 (AOX1) gene in recombinant protein expression in *Pichia pastoris*. Gene. 2012;496:118–27. 22285974 10.1016/j.gene.2012.01.006 PMC3464313

[18] Portela RM, Vogl T, Ebner K, Oliveira R, Glieder A. *Pichia pastoris* alcohol oxidase 1 (AOX1) core promoter engineering by high resolution systematic mutagenesis. Biotechnol J. 2018;13:1700340. 10.1002/biot.201700340 29125227

[19] Takagi S, Tsutsumi N, Terui Y, Kong X, Yurimoto H, Sakai Y. Engineering the expression system for *Komagataella phaffii* (*Pichia pastoris*): an attempt to develop a methanol-free expression system. FEMS Yeast Res. 2019. 10.1093/femsyr/foz059. 31408151 10.1093/femsyr/foz059 PMC6736287

[20] Haghighi Poodeh S, Ranaei Siadat SO, Arjmand S, Khalifeh Soltani M. Improving AOX1 promoter efficiency by overexpression of Mit1 transcription factor. Mol Biol Rep. 2022;49:9379–86. 36002652 10.1007/s11033-022-07790-7

[21] Shen S, Sulter G, Jeffries TW, Cregg JM. A strong nitrogen source-regulated promoter for controlled expression of foreign genes in the yeast *Pichia pastoris*. Gene. 1998;216:93–102. 9714758 10.1016/S0378-1119(98)00315-1

[22] Stadlmayr G, Mecklenbräuker A, Rothmüller M, Maurer M, Sauer M, Mattanovich D, Gasser B. Identification and characterisation of novel *Pichia pastoris* promoters for heterologous protein production. J Biotechnol. 2010;150:519–29. 20933554 10.1016/j.jbiotec.2010.09.957

[23] Liang S, Zou C, Lin Y, Zhang X, Ye Y. Identification and characterization of P GCW14: a novel, strong constitutive promoter of *Pichia pastoris*. Biotech Lett. 2013;35:1865–71. 10.1007/s10529-013-1265-8 23801118

[24] Waterham HR, Digan ME, Koutz PJ, Lair SV, Cregg JM. Isolation of the *Pichia pastoris* glyceraldehyde-3-phosphate dehydrogenase gene and regulation and use of its promoter. Gene. 1997;186:37–44. 9047342 10.1016/S0378-1119(96)00675-0

[25] Zhang AL, Luo JX, Zhang TY, Pan YW, Tan YH, Fu CY, Tu Fz. Recent advances on the GAP promoter derived expression system of *Pichia pastoris*. Mol Biol Rep. 2009;36:1611–9. 18781398 10.1007/s11033-008-9359-4

[26] Vogl T. Engineering of promoters for gene expression in *Pichia pastoris*. In: Mapelli V, Bettiga M, editors. Yeast metabolic engineering: methods and protocols. New York: Springer; 2022. p. 153–77. 10.1007/978-1-0716-2399-2_10 35781205

[27] Qin X, Qian J, Yao G, Zhuang Y, Zhang S, Chu J. GAP promoter library for fine-tuning of gene expression in *Pichia pastoris*. Appl Environ Microbiol. 2011;77:3600–8. 21498769 10.1128/AEM.02843-10 PMC3127614

[28] Ata Ö, Prielhofer R, Gasser B, Mattanovich D, Çalık P. Transcriptional engineering of the glyceraldehyde-3-phosphate dehydrogenase promoter for improved heterologous protein production in *Pichia pastoris*. Biotechnol Bioeng. 2017;114:2319–27. 28650069 10.1002/bit.26363

[29] Nguyen Ba AN, Pogoutse A, Provart N, Moses AM. NLStradamus: a simple hidden markov model for nuclear localization signal prediction. BMC Bioinform. 2009;10:1–11. 10.1186/1471-2105-10-202 PMC2711084 19563654

[30] Kosugi S, Hasebe M, Tomita M, Yanagawa H. Systematic identification of cell cycle-dependent yeast nucleocytoplasmic shuttling proteins by prediction of composite motifs. Proc Natl Acad Sci. 2009;106:10171–6. 19520826 10.1073/pnas.0900604106 PMC2695404

[31] Rey S, Acab M, Gardy JL, Laird MR, DeFays K, Lambert C, Brinkman FS. PSORTdb: a protein subcellular localization database for bacteria. Nucl Acid Res. 2005;33:D164–8. 10.1093/nar/gki027 PMC539981 15608169

[32] Urbinati CR, Gonsalvez Gb Fau - Aris JP, Aris Jp Fau - Long RM, Long RM. Loc1p is required for efficient assembly and nuclear export of the 60S ribosomal subunit. 10.1007/s00438-006-0151-7 16871394

[33] Maxon ME, Herskowitz I. Ash1p is a site-specific DNA-binding protein that actively represses transcription. Proc Natl Acad Sci. 2001;98:1495–500. 11171979 10.1073/pnas.98.4.1495 PMC29285

[34] Mat Nanyan NSb, Watanabe D, Sugimoto Y, Takagi H. Involvement of the stress-responsive transcription factor gene MSN2 in the control of amino acid uptake in *Saccharomyces cerevisiae*. FEMS Yeast Res. 2019. 10.1093/femsyr/foz052. 31328231 10.1093/femsyr/foz052

[35] Chatenay Lapointe M, Shadel GS. Repression of mitochondrial translation, respiration and a metabolic cycle-regulated gene, SLF1, by the yeast pumilio-family protein Puf3p. PLoS ONE. 2011;6: e20441. 21655263 10.1371/journal.pone.0020441 PMC3105058

[36] Turcotte B, Liang XB, Robert F, Soontorngun N. Transcriptional regulation of nonfermentable carbon utilization in budding yeast. FEMS Yeast Res. 2009;10:2–13. 19686338 10.1111/j.1567-1364.2009.00555.x PMC5003605

[37] Kim D, Song JY, Hahn JS. Improvement of glucose uptake rate and production of target chemicals by overexpressing hexose transporters and transcriptional activator Gcr1 in *Saccharomyces cerevisiae*. Appl Environ Microbiol. 2015;81:8392–401. 26431967 10.1128/AEM.02056-15 PMC4644637

[38] Ravi C, Gowsalya R, Nachiappan V. Impaired GCR1 transcription resulted in defective inositol levels, vacuolar structure and autophagy in* Saccharomyces cerevisiae*. Curr Genet. 2019;65:995–1014. 30879088 10.1007/s00294-019-00954-2

[39] Chang YL, Tseng SF, Huang YC, Shen ZJ, Hsu PH, Hsieh MH, Yang CW, Tognetti S, Canal B, Subirana L. Yeast Cip1 is activated by environmental stress to inhibit Cdk1–G1 cyclins via Mcm1 and Msn2/4. Nat Commun. 2017;8:1–14. 28676626 10.1038/s41467-017-00080-y PMC5496861

