# Supplementary material for: Novel transcriptional regulation of the GAP promoter in Pichia pastoris towards high expression of heterologous proteins
Source: Microb Cell Fact. 2024 Jul 24;23:206. doi: 10.1186/s12934-024-02435-9 (PMC11267847; doi:10.1186/s12934-024-02435-9)
Supplement: Supplementary file 1 — Supplementary material 1. [file 12934_2024_2435_MOESM1_ESM.docx]

**Supporting information**

**1. Primers used in this study**

**Table S1.** Primer information

| primer | sequence | purpose |
| --- | --- | --- |
| 1-F | ACTTAGTTTCTTCGAATTCGCGGCCGCTTCTAGAATGGAACCAGCCTTTAACTACG | For amplification of *E4* gene |
| 1-R | GTTTCTTCCTGCAGCGGCCGCTACTAGTTTAATGATGATGATGATGATGAG |  |
| 2-F | ACTTACGCGGATCCGCGATGAGCCATATTCAAC | For amplification of *Kan* gene |
| 2-R | ACTTACCGGAATTCCGGTTAGAAAAACTCATCGAG |  |
| 3-F | ACTTAGTTTCTTCCTAGAGCTGAAAAATAACAGTTATT | For amplification of *pGAP* gene |
| 3-R | ACTTAGTTTCTTCATCTTCTCAAGTTGTCGTTAAAAGTCGTT |  |
| 4-F | ACTTACCGGAATTCCGGATGAATGCATCTAGTATCC | For amplification of *Hot1* gene |
| 4-R | CCTGTAGGGGGCTCAATTCCACCGAATCCATTTGAC |  |
| 5-F | CGGTGGAATTGAGCCCCCTACAGGGTATTCGATTGTGT |  |
| 5-R | ACTTATTTGCGGCCGCTTTACTACAAGTCATAATT |  |
| 6-F | ACTTACCGGAATTCCGGATGGTAGACAAAGCTACTAC | For amplification of *Gsm1* gene |
| 6-R | ACTTATTTGCGGCCGCTTTATCATGTCAAGATGGGGAG |  |
| 7-F | ACTTACCGGAATTCCGGATGTCTACAACAAAACCAAT | For amplification of *Msn2* gene |
| 7-R | TACGCCCTGGCTAGGAAGAACAGAGTTCGAACAGAT |  |
| 8-F | CTGTTCTTCCTAGCCAGGGCGTAACTTCGCAACATAGCT |  |
| 8-R | ACTTATTTGCGGCCGCTTTATCACTGCTTACGGTGAGTACGT |  |
| 9-F | ACTTACGCGGATCCGCGATGCCTCGCAATAAGACT | For amplification of *Loc1* gene |
| 9-R | ACTTACCGGAATTCCGGTTAAGCAAATGACACAGATT |  |
| 10-F | ACTTACGAGCTCGACCATTCGTCTGTGGTTGTTTAACT | For amplifying the upstream homologous arm of *Hot1* gene |
| 10-R | ACTTACGCGGATCCGCGGGACTGCTCAGGCCATCAAT |  |
| 11-F | ACTTATTTGCGGCCGCTTTACTCCCAAATACAAATCCTAC | Used to amplify the downstream homologous arm of *Hot1* gene |
| 11-R | ACTTAGTTTCTTCGTCGACTCTTGAGAGGGATTCAATAC |  |
| 12-F | ACTTACGAGCTCGCTGGTTTAAGATTGGCAAT | For amplifying upstream homologous arm of *Gsm1* gene |
| 12-R | ACTTACGCGGATCCGCGTCGATCGAAGACTTGAGT |  |
| 13-F | ACTTACCGGAATTCCGGACATATTAAACACTACTT | For amplifying downstream homologous arm of *Gsm1* gene |
| 13-R | ACTTATTTGCGGCCGCTTTAAACGTCGTCAATTTTGATAT |  |
| 14-F | ACTTACGAGCTCGGCTCAGTGGCTTTCCATCTGTT | For amplifying the upstream homologous arm of *Msn2* gene |
| 14-R | CTTACGCGGATCCGCGTCTAGTTAATCGCAAAC |  |
| 15-F | ACTTATTTGCGGCCGCTTTAAGGAATTGGGAAGGTTTTATATT | Used to amplify downstream homologous arm of *Msn2* gene |
| 15-R | ACTTAGTTTCTTCGTCGACTCTTTTATTGAGCCTGTGTC |  |
| 16-F | ACTTACGAGCTCGACAGGCAAATCATTCA | For amplifying upstream homologous arm of *Loc1* gene |
| 16-R | ACTTACGCGGATCCGCGCGTATTTAGCTACAATC |  |
| 17-F | ACTTACCGGAATTCCGGTCACTAACTGTATACT | Used to amplify downstream homologous arm of *Loc1* gene |
| 17-R | ACTTATTTGCGGCCGCTTTAACAATCAAGAGGACATACTT |  |
| 18-F | TGACTTGGTCCGCTTCTTCC | QPCR for *E4* gene |
| 18-R | TGGTTCACATCCGGTCAAGG |  |
| 19-F | AGATCTTTTTTGTAGAAATGTCTTGGTGTCCTCGTCC | Biotin-labeled for amplification of *pGAP* probe |
| 19-R | GTAAAATTTCCTAGGGACGGTAACGGGCGGT |  |
| 20-F | AGATCTTTTTTGTAGAAATGTCTTGGTGTCCTCGTCC | Biotin-labeled for amplification of pGAP-AB probe |
| 20-R | GTAAAATTTCCTAGGGACGGTAACGGGCGGT |  |
| 21-F | TCTCTTCCCTTCTCTCTCCTTCCACCGC | Biotin-labeled for amplification of pGAP-BC probes |
| 21-R | CATGCGTCCGCCCGCTATTATTGCC |  |
| 22-F | AGCAGCCCAGGGATGGAAAAGTC | Biotin-labeled for amplification of pGAP-CD probes |
| 22-R | CGTTTCGAAATAGTTGTTCAATTGATTGAAATAGGG |  |
| 23-F | TGGCTTTCCATCTGTTTGATAGACAGAAGATCTCGAGGTAT | For constructing C100 complement expression boxes |
| 23-R | TGCTAAGGCTGGGACTACTTATTTGTGTTCTAGTTAATCGCAAACAAACCCAAATAATCA |  |
| 24-F | ATGACACAAATAAGTAGTCCCAGCCTTAGCACCC |  |
| 24-R | TCTTTTATTGAGCCTGTGTCCTCTAAGGCAACCAACAATTT |  |

**2. HDOCK docking results**

**Table S2.1** Docking results of Loc1p and pGAP

| **Rank** | **Docking Score** | **Confidence Score** | **Receptor interface residue(s)** | **Model** |
| --- | --- | --- | --- | --- |
| **1** | -193.24 | 0.7037 | A:174-179 S:308-312 | 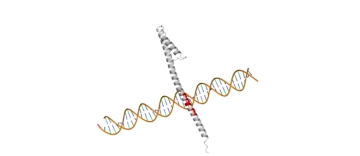 |
| **2** | -190.55 | 0.6923 | A:44-49 S:438-442 | 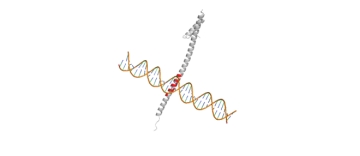 |
| **3** | -190.54 | 0.6923 | A:259-263 S:223-228 | 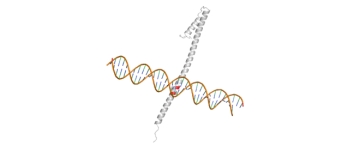 |
| **4** | -187.33 | 0.6784 | A:252-257 S:223-230 | 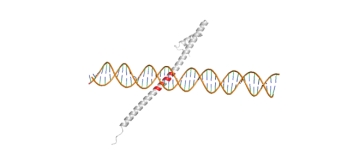 |
| **5** | -186.41 | 0.6744 | A:80-85 S:402-407 | 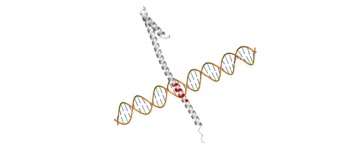 |
| **6** | -185.35 | 0.6697 | A:259-263 S:223-228 | 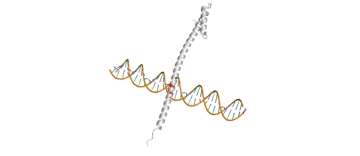 |
| **7** | -184.5 | 0.666 | A:442-447 S:40-45 | 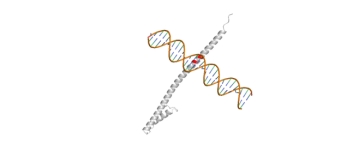 |
| **8** | -183.28 | 0.6605 | A:24-31 S:449-456 | 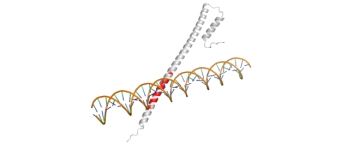 |
| **9** | -183.01 | 0.6593 | A:27-33 S:447-454 | 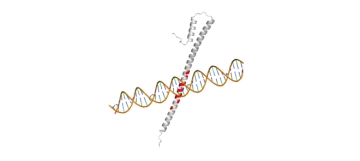 |
| **10** | -182.94 | 0.659 | A:116-123 S:359-364 | 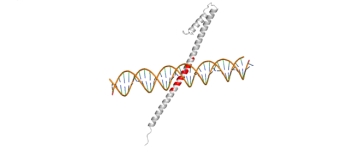 |

**Table S2.2** Docking results of C100 and pGAP

| **Rank** | **Docking Score** | **Confidence Score** | **Receptor interface residue(s)** | **Interface residues** |
| --- | --- | --- | --- | --- |
| **1** | -278.54 | 0.929 | A:229-235 S:245-253 | 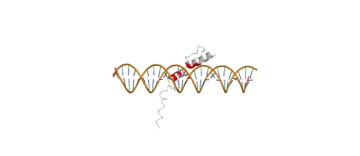 |
| **2** | -277.51 | 0.9276 | A:238-245 S:247-255 | 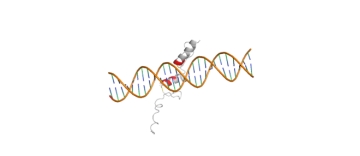 |
| **3** | -276.99 | 0.9269 | A:438-445 S:37-44 | 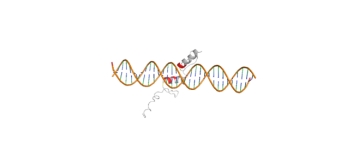 |
| **4** | -276.86 | 0.9267 | A:38-45 S:437-443 | 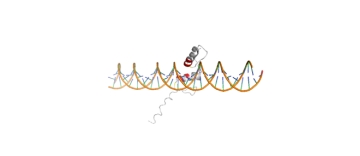 |
| **5** | -276.77 | 0.9266 | A:196-201 S:279-286 | 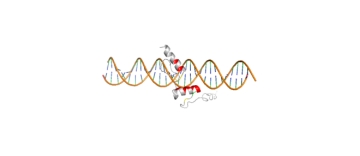 |
| **6** | -276.3 | 0.9259 | A:151-156 S:323-331 | 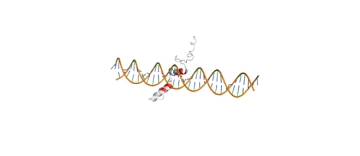 |
| **7** | -275.51 | 0.9249 | A:156-162 S:318-326 | 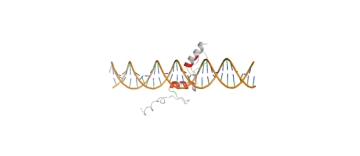 |
| **8** | -275.36 | 0.9246 | A:159-165 S:315-323 | 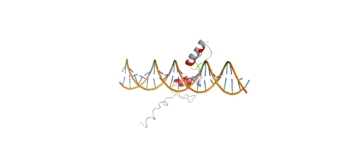 |
| **9** | -274.35 | 0.9232 | A:159-165 S:315-323 | 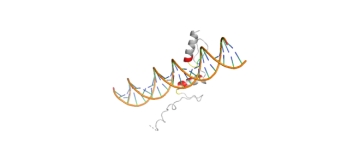 |
| **10** | -274.06 | 0.9228 | A:210-217 S:264-271 | 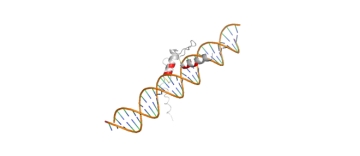 |

**Table S2.3** Docking results of Gsm1p and pGAP

| **Rank** | **Docking Score** | **Confidence Score** | **Receptor interface residue(s)** | **Interface residues** |
| --- | --- | --- | --- | --- |
| **1** | -235.8 | 0.8476 | A:88-96  S:389-399 | 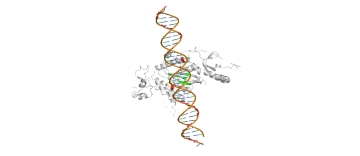 |
| **2** | -234.29 | 0.8437 | A:195-206 S:281-206 | 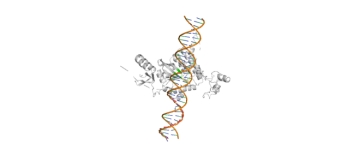 |
| **3** | -233.46 | 0.8415 | A:393-403 S:84-91 | 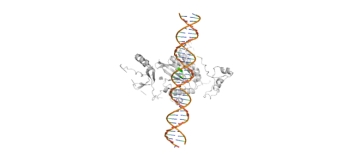 |
| **4** | -231.57 | 0.8364 | A:246-270 S:217-241 | 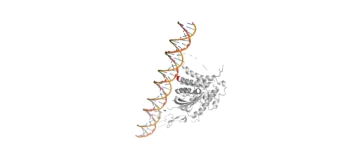 |
| **5** | -230.37 | 0.8331 | A:139-159 S:327-350 | 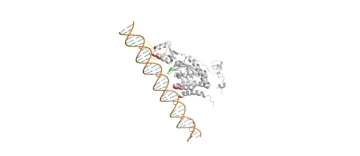 |
| **6** | -225.65 | 0.8195 | A:341-349 S:136-146 | 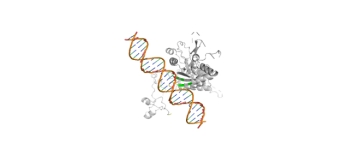 |
| **7** | -224.4 | 0.8158 | A:67-77 S:410-418 | 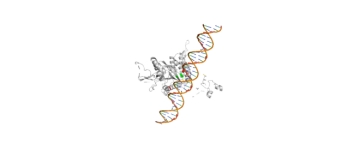 |
| **8** | -220.86 | 0.8049 | A:316/317/324-327 S:152-154/161-165/172-175 | 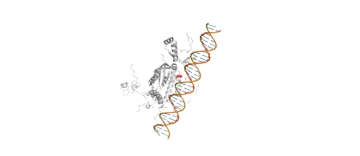 |
| **9** | -216.62 | 0.7912 | A:245-247/254-258/264-269 S:213/223-234 | 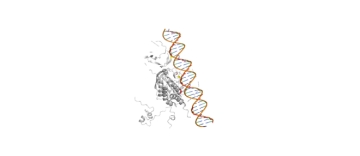 |
| **10** | -215.68 | 0.7881 | A:398-409 S:78-86 | 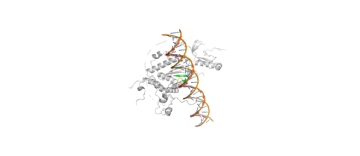 |

**Table S2.4** Docking results of Hot1p and pGAP

| **Rank** | **Docking Score** | **Confidence Score** | **Receptor interface residue(s)** | **Interface residues** |
| --- | --- | --- | --- | --- |
| **1** | -260.9 | 0.9019 | A:359-376 S:106-123 | 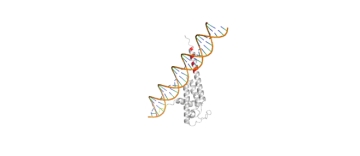 |
| **2** | -259.74 | 0.8998 | A:148-166 S:316-333 | 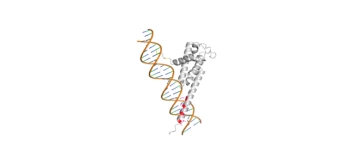 |
| **3** | -253.32 | 0.8876 | A:376-378/385-392 S;89-94/101-108 | 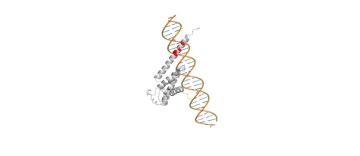 |
| **4** | -253.18 | 0.8873 | A:312-317/324-330 S:152-156/162-169 | 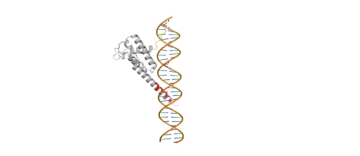 |
| **5** | -252.22 | 0.8854 | A:349-354/361-367 S:115-119/125-132 | 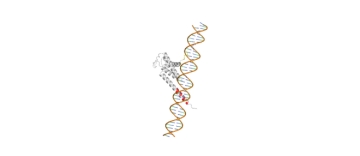 |
| **6** | -251.55 | 0.884 | A:402-407/414-420 S:62-65/72-79 | 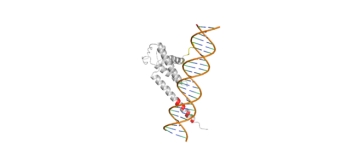 |
| **7** | -251.3 | 0.8835 | A:345-350/356-363 S:119-124/130-137 | 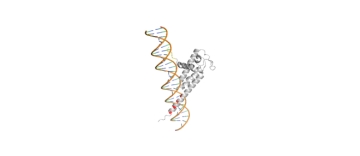 |
| **8** | -249.79 | 0.8804 | A:251-255/261-268 S:213-218/225-231 | 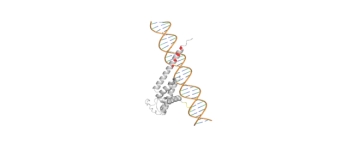 |
| **9** | -249.46 | 0.8797 | A:3498-365 S:117-121/128-135 | 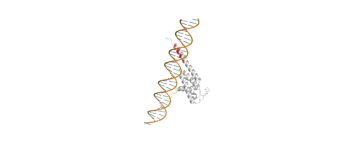 |
| **10** | -248.06 | 0.8767 | A:132-137/144-150 S:332-336/342-349 | 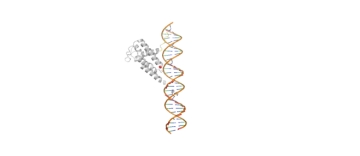 |

1. **Design of EMSA experimental groups**

**Table S3.** Design of experimental groups

| group | biotin-labeled DNA(ng) | binding buffer(µL) | transcription factors(µg) | unlabeled DNA(µg) | unlabeled irrelevant DNA(µg) | target protein corresponding to antibodies(µg) |
| --- | --- | --- | --- | --- | --- | --- |
| blank control group | 50 | 2 | - | - | - | - |
| experimental group | 50 | 2 | 2 | - | - | - |
| cold-probe competition experimental group | 50 | 2 | 2 | 5 | - | - |
| irrelevant cold-probe competition experimental group | 50 | 2 | 2 | - | 5 | - |
| supershift experimental group | 50 | 2 | 2 | - | - | 4 |

**4. Results of SMART domain analysis**

**Table S4.** Structure prediction results of transcription factors

| Transcription factor | Domain | Domain localization | Division of transcription factors | Analysis results of SMART |
| --- | --- | --- | --- | --- |
| Loc1p | Coiled coil | 113-184 | LN:100 aa at N-terminal  LC：113 aa at C-terminal | 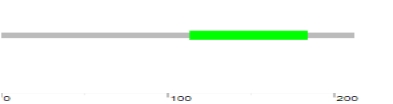 |
| Msn2p | ZnF_C2H2 | 242-265  271-293 | C100:100 aa at C-terminal | 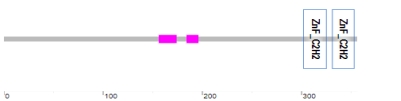 |
| Gsm1p | GAL4-like Zn(II)2Cys6 (or C6 zinc) | 23-68 | GN:234 aa at N-terminal  GC：248 aa at C-terminal | 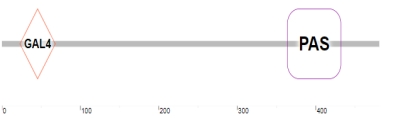 |
|  | putative active site | 364-432 |  |  |
| Hot1p | coiled coil | 111-131  207-234 | HN:200 aa at N-terminal  HC：203 aa at C-terminal | 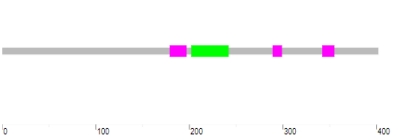 |
|  | coiled coil | 178-196  288-298  341-354 |  |  |
|  | GCR1_C | 304-381 |  |  |

**5. Construction of recombinant plasmid**


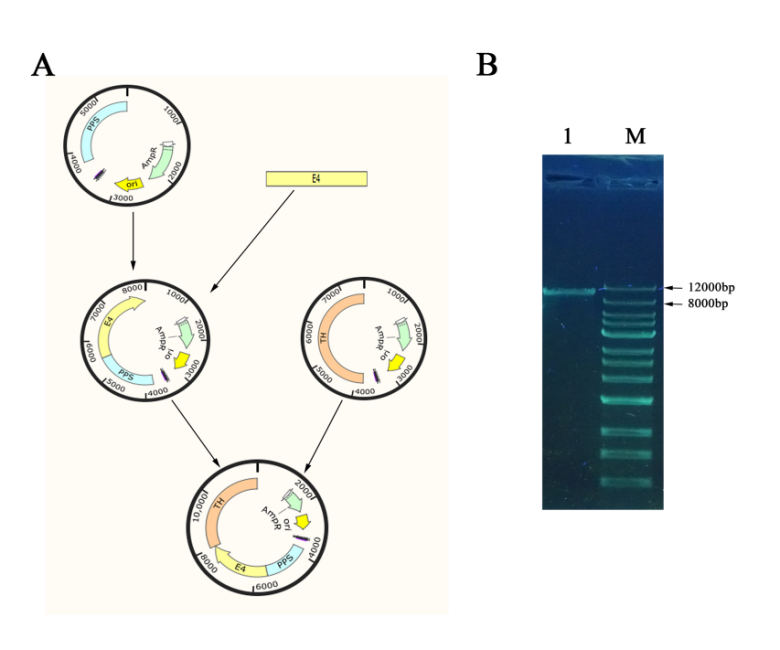


**Figure S1.** Construction of recombinant plasmid. (A) Roadmap of plasmid construction. (B) Plasmid verification map 1: *pAOX1-pGAP-ss1-E4-Taox-PgHT- pBAN* Plsamid (molecular mass:11772 bp; the vectors contained dual promoters:the pAOX1 promoter was used during methanol induction, and the pGAP promoter was used when glycerol or glucose was used as the carbon source). The band size was in accordance with the expected value by single enzyme digestion, which proved that the vector construction was complete.

**6. Prediction results of transcription factor binding sites**

**
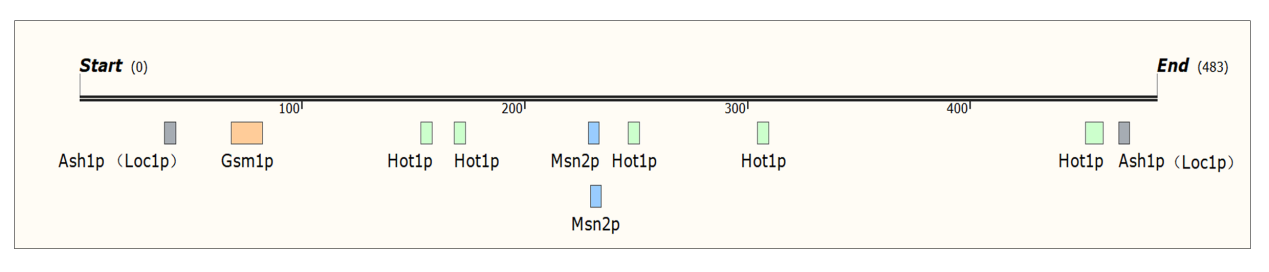
Figure S2.** Prediction results of transcription factor binding sites

**7. Structural model of the transcription factors**


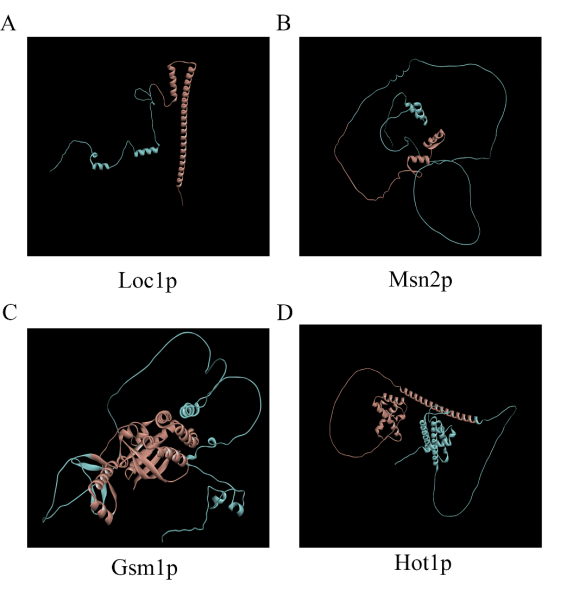


**Figure S3.** Models showing structures of the transcription factors (blue color indicates the N-terminal; red color indicates the C-terminal). Structures of the four transcription factors have been predicted using the AlphaFold model.

**8. Construction of transcription factor** **knockout or overexpression strains**

**8.1 Construction of transcription factor overexpression vector**

The pGAP sequence was amplified using 3-F/R and ligated to *ppic3.5k* through the *Sac*I / *Bam*HI restriction site. The *Hot1* gene was amplified by overlapping PCR using 4-F/R and 5-F/R and ligated to the *pGAP-ppic3.5k* vector by *Eco*RI / *Not*I. The *Gsm1 / Msn2 / Loc1* genes were amplified by PCR using 6-F/R, 7/8-F/R and 9-F/R, respectively, and ligated to the *pGAP-ppic3.5k* vector by *Eco*RI / *Not*I. The plasmid map is as follows :


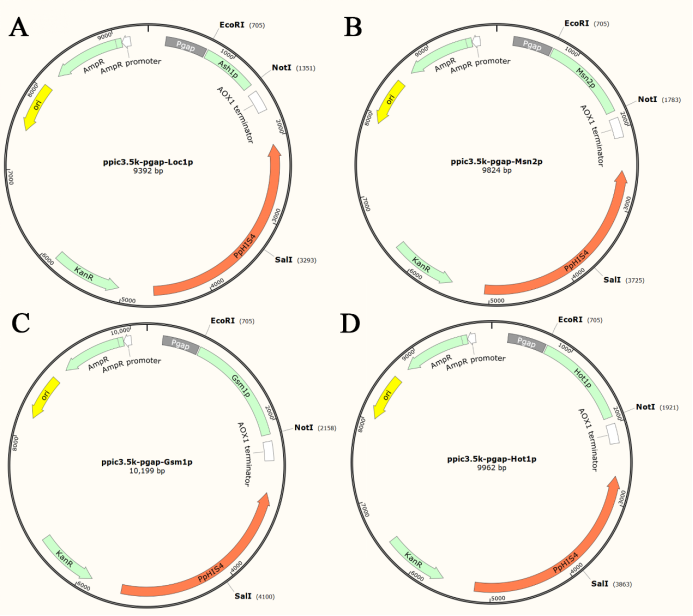


**Figure S4**. Construction of transcription factor overexpression vector. (A) *Loc1* overexpression vector (B) *Msn2* overexpression vector (C) *Gsm1* overexpression vector (D) *Hot1* overexpression vector.

**8.2 Construction of transcription factor knockout expression cassette**

The upstream and downstream homologous arms of *Loc1* were amplified using 17-F/R and 18-F/R to construct the knockout expression cassette. According to this method, three other transcription factor knockout expression cassettes were constructed. The plasmid map is as follows:


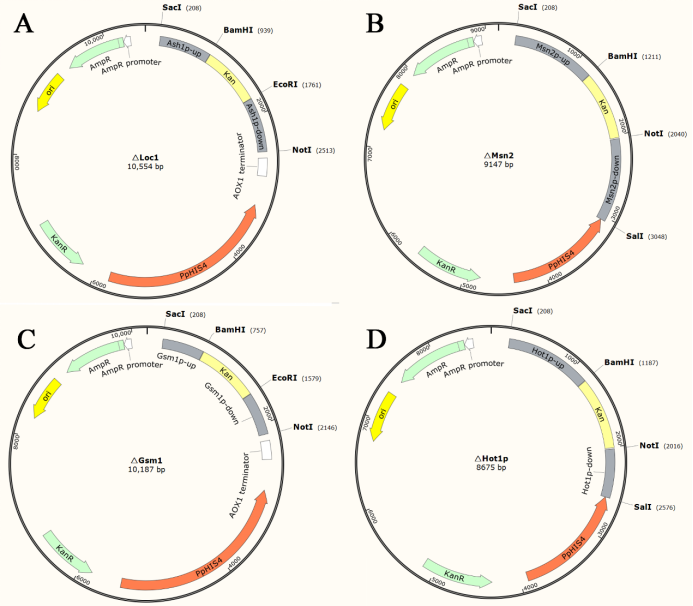


**Figure S5.** Transcription factor knockout vector construction. (A) *Loc1* knockout vector (B) *Msn2* knockout vector (C) *Gsm1* knockout vector (D) *Hot1* knockout vector.

1. **Transcription factor expression**

The transcription factor-containing *E. Coli* was cultivated in 300 mL of LB medium (include 50 μg/mL Kan) for a duration of 200 rpm and 37 °C, until the bacteria reached an OD600 of 0.8. The medium received an addition of IPTG (final concentration: 1 mmol/L) for a 6-hour induction of expression. Centrifugation at 6000 g for 5 minutes was used to gather the cells, and they were then three times washed with an equivalent volume of PBS (pH 7.4). Ultimately, the cells underwent ultrasonic crushing (crushing conditions: total time: 30 min; crushing time: 5s; interval time: 7s; temperature: 4 °C; power: 300W) after being resuspended in 50 mL PBS. The target protein was found in the broken supernatant, which was obtained by centrifuging at 8000 g for 10 min at 4 °C. His-tag antibody detection was used to do a Western blot verification of transcription factors.


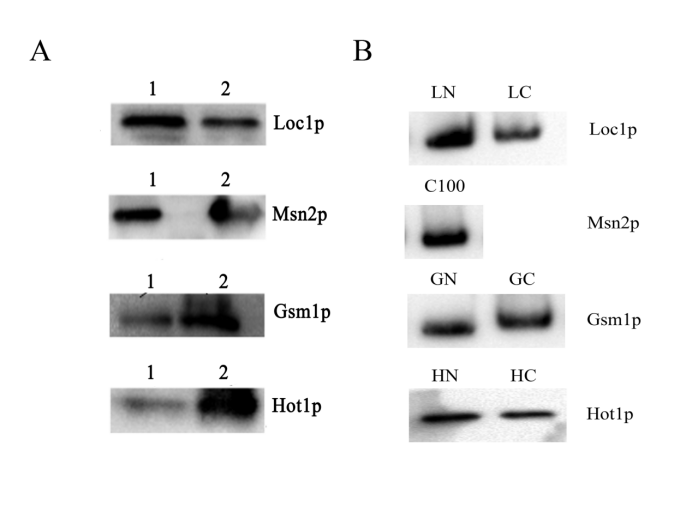


**Figure S6.** Transcription factor expression results. (A) Protein expression results of transcription factor : 1 is the supernatant of cell disruption ; 2 is broken sediment.

(B) Protein expression results of different domains of transcription factor

**10. Diagram depicting the** **partitioning of pGAP**


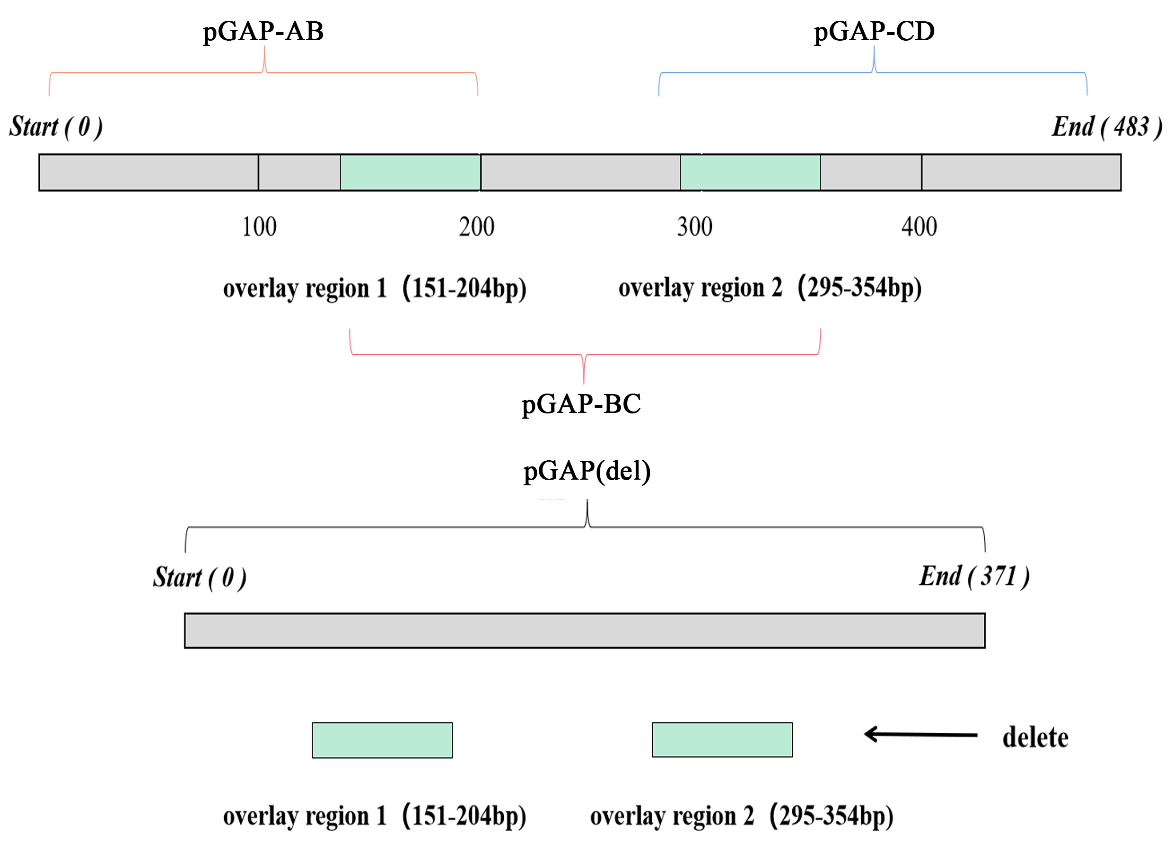


**Figure S7.** Diagram depicting the partitioning of pGAP.

**11. Prediction results of NLS**


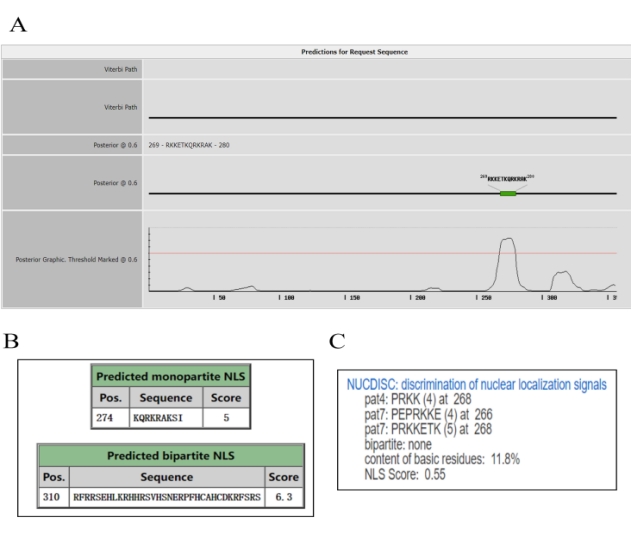


**Figure S8.** Prediction results of NLS. (A) Results predicted by NLStradamus. (B) Results predicted by cNLS Mapper. (C) Results predicted by CPSORT II.

**12. Results of protein interactions**


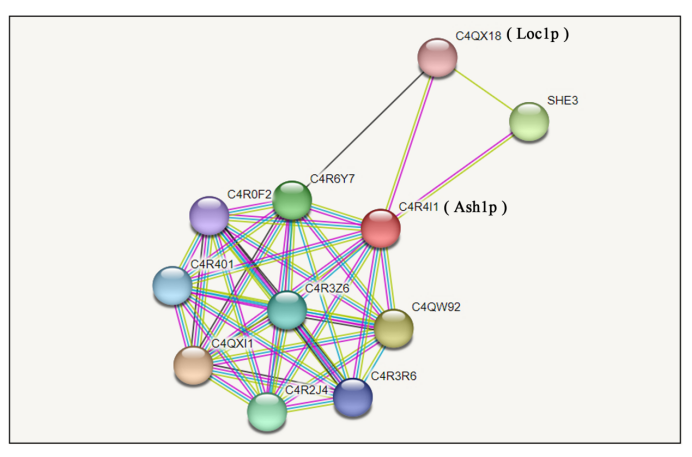


**Figure S9.** The results of protein interactions between Loc1p and Ash1p

**13.UV-induced mutagenesis directed evolution**

The cells were cultured in YPG medium at 28 °C until the number of cells reached 10^7^–10^8^ cells / mL. These were then washed twice with 1 × PBS and 1 mL 0.1 M β-mercaptoethanol was added. The culture was incubated in a metal bath at 30 °C for 20 min, then resuspended in 1 mL 1 × PBS and washed three times. Then, 1 mL snailase solution was added and mixed, and the mixture was digested in a metal bath at 28 °C for 1.5 h. The digested bacteria were resuspended and washed twice with 1 mL 0.6 M KCl solution to obtain *Pichia pastoris* *SMD1168* protoplasts.

The prepared protoplasts were diluted to 10^-3^ and spread on the hyperosmotic solid medium. The protoplasts were irradiated for 0–70 s at 30 cm under a UV lamp in a dark environment (15 W, 265 nm). After irradiation, they were cultured at 30 °C in dark for 72–120 h. Three parallels were set for each irradiation time point. The strains with a lethality rate of 70–80 % under ultraviolet irradiation were selected and screened in CMC-Na medium. Strains with fiber degradation ability formed a clear transparent circle on the medium, and the size of the transparent circle was related to the activity of the cellulase secreted by the strain. The strains with a larger R value than the original strain ( *EX_6_* ) ( R = d2 / d1, where d1 is the colony diameter and d2 is the hydrolysis circle size ) were selected and cultured for several generations to detect genetic stability. Then, the potential high expression strains with genetic stability were selected and inoculated in 5 mL YPG medium at 28 °C. They were then placed in a shaking incubator at 200 rpm for 72 h. Protein expression was identified using SDS-PAGE. The screened high expression strains were subjected to three rounds of cumulative UV mutagenesis-directed evolution, and the final screening results are as follows: The strains screened in the first round were recorded as *EX_6-34_*, those in the second round as *EX_6-34-16_*, and the finally screened high expression strains as *EX_6-34-16-15_*).

1. **The culture conditions and the determination of relative content and relative amounts**

The *P. pastoris* containing the reporter protein gene were cultured in YPG medium (1% Yeast Extract, 2% soy peptone, 1% glycerol) at 30°C and 200 rpm for approximately 18 hours until reaching an OD600 of 1.6 to 2.0. The cells were then cultured in 100 mL of YPG medium or BMMY medium (1 % Yeast Extract, 2 % soy peptone, 1 % methanol) with 0.1% inoculum at 30°C and 200 rpm for 72 hours. Biomass was determined by measuring the OD600 using an ultraviolet spectrophotometer.

As the target protein is expressed extracellularly, the protein content in the supernatant can accurately reflect the expression level of the reported protein for each strain when the biomass is the same. The same biomass strain's culture supernatant was exposed to western blot verification, and grayscale scanning was performed to compare the protein expression levels. The mean values of the results of three repeated experiments are depicted, and the relative standard deviations are shown using error bars.
